# The neuroendocrine transition in prostate cancer is dynamic and dependent on ASCL1

**DOI:** 10.1038/s43018-024-00838-6

**Published:** 2024-10-11

**Authors:** Rodrigo Romero, Tinyi Chu, Tania J. González Robles, Perianne Smith, Yubin Xie, Harmanpreet Kaur, Sara Yoder, Huiyong Zhao, Chenyi Mao, Wenfei Kang, Maria V. Pulina, Kayla E. Lawrence, Anuradha Gopalan, Samir Zaidi, Kwangmin Yoo, Jungmin Choi, Ning Fan, Olivia Gerstner, Wouter R. Karthaus, Elisa DeStanchina, Kelly V. Ruggles, Peter M. K. Westcott, Ronan Chaligné, Dana Pe’er, Charles L. Sawyers

**Affiliations:** 1 Human Oncology and Pathogenesis Program, Memorial Sloan Kettering Cancer Center, New York, NY USA; 2 Program for Computational and Systems Biology, Sloan Kettering Institute, Memorial Sloan Kettering Cancer Center, New York, NY USA; 3 Institute of Systems Genetics, Department of Precision Medicine, NYU Grossman School of Medicine, New York, NY USA; 4 Department of Biochemistry and Molecular Pharmacology, NYU Grossman School of Medicine, New York, NY USA; 5 Department of Pathology, Memorial Sloan Kettering Cancer Center, New York, NY USA; 6 Antitumor Assessment Core Facility, Memorial Sloan Kettering Cancer Center, New York, NY USA; 7 Molecular Cytology Core Facility, Memorial Sloan Kettering Cancer Center, New York, NY USA; 8 Department of Genitourinary Oncology, Memorial Sloan Kettering Cancer Center, New York, NY USA; 9 Department of Biomedical Sciences, Korea University College of Medicine, Seoul, Korea; 10 Cold Spring Harbor Laboratory, Cold Spring Harbor, NY USA; 11 Howard Hughes Medical Institute, Chevy Chase, MD USA

**Keywords:** Cancer, Cancer models

## Abstract

Lineage plasticity is a hallmark of cancer progression that impacts therapy outcomes, yet the mechanisms mediating this process remain unclear. Here, we introduce a versatile in vivo platform to interrogate neuroendocrine lineage transformation throughout prostate cancer progression. Transplanted mouse prostate organoids with human-relevant driver mutations (*Rb1*^*−*/*−*^; *Trp53*^*−*/*−*^; *cMyc*^*+*^ or *Pten*^*−*/*−*^; *Trp53*^*−*/*−*^; *cMyc*^*+*^) develop adenocarcinomas, but only those with *Rb1* deletion advance to aggressive, ASCL1^+^ neuroendocrine prostate cancer (NEPC) resistant to androgen receptor signaling inhibitors. Notably, this transition requires an in vivo microenvironment not replicated by conventional organoid culture. Using multiplexed immunofluorescence and spatial transcriptomics, we reveal that ASCL1^+^ cells arise from KRT8^+^ luminal cells, progressing into transcriptionally heterogeneous ASCL1^+^;KRT8^−^ NEPC. *Ascl1* loss in established NEPC causes transient regression followed by recurrence, but its deletion before transplantation abrogates lineage plasticity, resulting in castration-sensitive adenocarcinomas. This dynamic model highlights the importance of therapy timing and offers a platform to identify additional lineage plasticity drivers.

## Abstract (web summary)

Sawyers and colleagues describe an in vivo platform used to explore the dynamics and key factors of neuroendocrine lineage transformation. They find that Ascl1 depletion blocks plasticity and leads to cancer that is sensitive to castration.

## Main

Prostate cancer is the leading cause of cancer death globally in men^1^. Survival has improved with next-generation androgen receptor signaling inhibitors, but patients eventually progress to castration-resistant prostate cancer^2^. Although men receiving androgen receptor signaling inhibitors live longer, an increasing fraction display features of lineage plasticity at relapse, characterized by reduced or absent expression of luminal lineage markers such as the androgen receptor (AR) and prostate-specific antigen^3,4^. In its extreme form, lineage plasticity manifests as a transition to neuroendocrine (NE) histology called NEPC, with expression of synaptophysin (SYP) and chromogranins^4^. Patients with NEPC often have soft tissue metastases (for example, liver) rather than bone, suggesting a role of the tumor microenvironment (TME) in this transition^5,6^. Similar lineage transitions are observed in other cancers treated with targeted therapies, for example, *EGFR*-, *ALK*- and *KRAS*^*G12C*^-mutant lung adenocarcinoma, underscoring the broad relevance of lineage plasticity in tumor progression and therapy resistance^7–11^.

The molecular details underlying these lineage transitions are poorly understood, owing to a shortage of model systems that accurately replicate these processes. Autochthonous models of prostate cancer have been valuable, but few capture the transition at all stages or are amenable to intervention in a timely and cost-effective manner^12–18^. Studies using prostate tumor cell line transplant models are easier to implement but limited in number and fail to replicate all transition stages observed in patients. To better understand NEPC and develop intervention strategies that curtail lineage plasticity, models that accurately reproduce the longitudinal molecular and morphologic features of these lineage transitions are needed.

Organoid technology has greatly expanded our ability to model epithelial biology, including prostate cancer^19,20^. Previously, we described a strategy to assess genetic drivers of prostate adenocarcinoma (PRAD), as well as cells of origin using mouse prostate organoids coupled with orthotopic transplantation^21^ (OT). Here, we optimize this approach into a robust platform for rapid, side-by-side assessment of cancer initiation and progression using multiple combinations of human-relevant cancer drivers in vivo. Using multiplexed spatial techniques, we detect isolated NE cells emerging from luminal epithelial cells, which subsequently evolve to fully penetrant NEPC, together with temporal changes within the TME, and perform functional perturbations that dramatically impact the lineage plasticity program.

## Results

### Organoid allelic series and tumor phenotype characterization

**Fig. 1:**
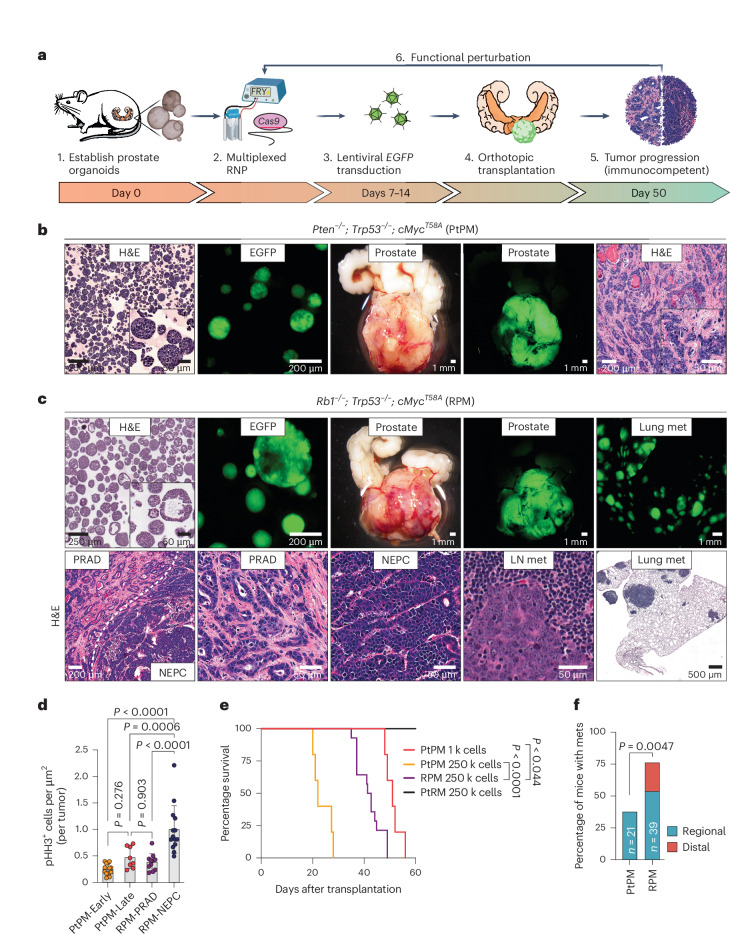
Rapid establishment of genetically defined prostate cancer with prostate organoids transplanted into immunocompetent syngeneic hosts. **a**, Schematic of timeline required to establish, propagate, edit and select for organoids harboring mutations in cancer-associated genes before transplantation into immunocompetent hosts for tumor establishment. **b**, Representative microscopy of *Pten*^*−*/*−*^; *Trp53*^*−*/*−*^; *cMyc*^*T58A*^ (PtPM) organoids, and stereoscopic and fluorescence images of OT prostate tumors with PRAD histology. Tumor images are representative of *n* = 22 mice. **c**, Representative microscopy of *Rb1*^*−*/*−*^; *Trp53*^*−*/*−*^; *cMyc*^*T58A*^ (RPM) organoids and stereoscopic and fluorescence images of OT prostate tumors and lung metastases (mets) (top). Representative histological assessment of RPM-PRAD and RPM-NEPC primary tumor or metastases histology at varying magnifications (bottom). Primary and metastatic microscopy and histology are representative of *n* = 25 mice. LN, lymph node (iliac). **d**, Phospho-histone H3 (Ser10; pHH3)-positive tumor cells per total tumor area (µm^2^). Each data point represents the average number of pHH3^+^ cells per individual tumor subset by tumor histology (PRAD versus NEPC) and experimental end point. PtPM-Early (<4 weeks), *n* = 14; PtPM-Late (>6 weeks), *n* = 8; RPM-PRAD, *n* = 11; and RPM-NEPC, *n* = 14 tumors. Statistics were derived using a one-way ANOVA with Tukey’s multiple comparisons correction. Error bars denote mean and s.d. **e**, Survival of mice transplanted with the indicated cell numbers of PtPM, RPM and *Pten*^*−*/*−*^; *Rb1*^*−*/*−*^; *cMyc*^*T58A*^ (PtRM) ex vivo edited organoids. PtPM 1k, *n* = 5; PtPM 250k, *n* = 5; RPM 250k, *n* = 14; and PtRM 250k, *n* = 8 mice. Statistics derived from the log-rank (Mantel–Cox) test for each pairwise comparison. **f**, Metastatic disease penetrance of the indicated organoid transplants. Regional metastases include dissemination into the iliac LNs. Distal metastases include dissemination to kidney, pancreas, liver or lungs. Statistics were derived from a two-sided Fisher’s exact test. The number of biological replicates is indicated within the figure. Scale bars are indicated within each panel. Source data

**Extended Data Fig. 1:**
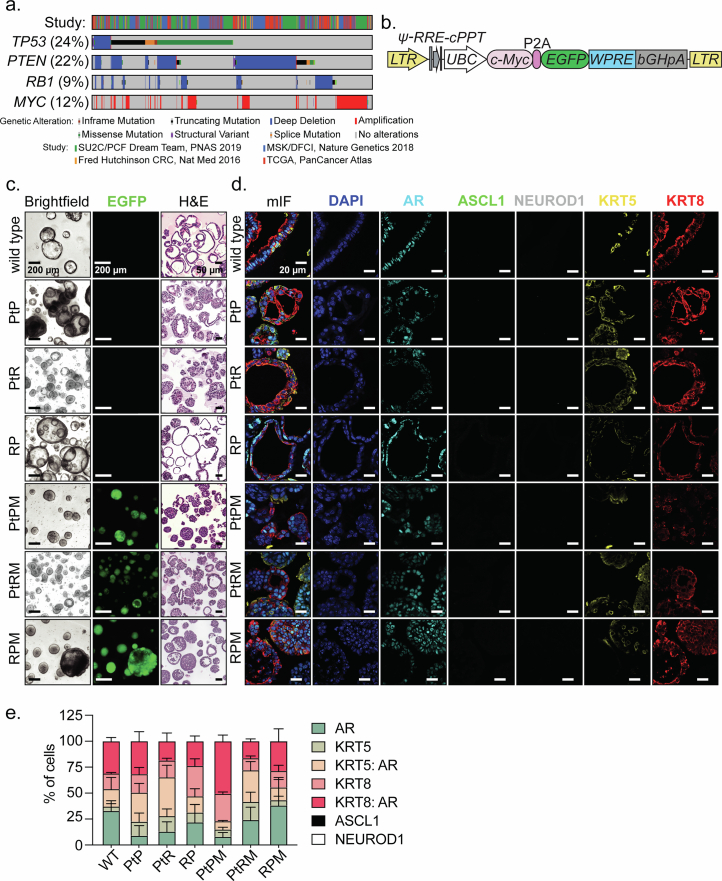
Engineering of clinically relevant mutations within an allelic mouse prostate organoid series. **a**. Oncoprint of the indicated genes frequently mutated in human primary prostate cancer. Data (*n* = 1633 patient samples) obtained from the studies indicated within the figure legend. **b**. Schematic of the lentiviral vector used within this study to overexpress *cMyc*^*T58A*^ transcriptionally linked to EGFP in organoids. **c**. Representative brightfield (left), GFP fluorescence (center), and hematoxylin & eosin (H&E) stains (right) of organoids harboring mutations in indicated tumor suppressors and oncogenes. Images are representative of *n* = 3 technical replicate organoid samples per genotype. H&E and GFP imaging were repeated independently twice with similar results. **d**. Representative confocal images of 7-plex mIF stains of organoids of the indicated proteins. Data are representative of *n* = 3 technical replicates per genotype. **e**. Percentage of unique cell types expressing the indicated markers in organoid culture. Data representative of *n* = 3 technical replicates and related to images in panel **d**. Error bars denote mean and s.d. All scale bars and pseudocolor legends indicated within the figure panel. Source data

We sought to develop a platform to interrogate prostate cancer drivers rapidly and comprehensively at larger scale compared to traditional genetically engineered mouse models (GEMMs), focusing on the need to dynamically model the PRAD-to-NEPC transition. Using multiplexed editing and lentiviral oncogene delivery, we established organoids with six combinations of cancer drivers selected based on their co-occurrence in human prostate cancer (Fig. 1a, Extended Data Fig. 1a–c and Supplementary Table 1; hereafter: *Pten*^*−*/*−*^; *Trp53*^*−*/*−*^ = PtP, *Rb1*^*−*/*−*^; *Trp53*^*−*/*−*^ = RP, *Pten*^*−*/*−*^; *Rb1*^*−*/*−*^ = PtR, *Pten*^*−*/*−*^; *Trp53*^*−*/*−*^; *cMyc*^+^ = PtPM, *Rb1*^*−*/*−*^; *Trp53*^*−*/*−*^; *cMyc*^+^ = RPM, *Pten*^*−*/*−*^; *Rb1*^*−*/*−*^; *cMyc*^+^ = PtRM). Consistent with previous work, histological assessment of edited mouse organoids grown in three-dimensional (3D) culture revealed a mixture of KRT5^+^ basal and KRT8^+^ luminal cells, with both populations staining for nuclear AR (ref. ^19^ and Extended Data Fig. 1d,e). All organoids lacked expression of NE transcription factors achaete-scute family bHLH transcription factor 1 (ASCL1) and neuronal differentiation 1 (NEUROD1), despite prolonged in vitro culture (Extended Data Fig. 1d,e).

**Extended Data Fig. 2:**
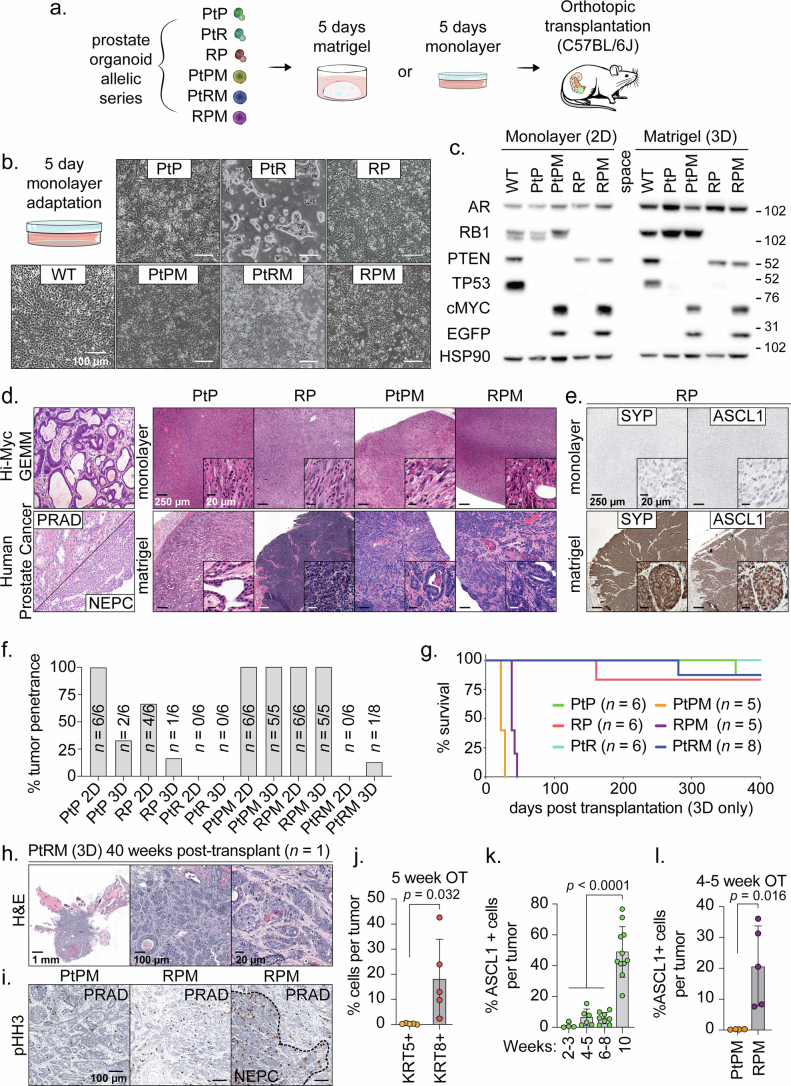
Transplantation of 3D, but not 2D, cultured mouse organoids maintain PRAD histology and marker profiles. **a**. Schematic representation of steps taken to establish OT prostate tumors in mice from edited organoids grown in matrigel (3D) or monolayer culture (2D) conditions. **b**. Representative brightfield images of the indicated organoids seeded in monolayer growth. Images taken 5 days post seeding. Images representative of *n* = 2 independent experiments with similar results. **c**. Western blot validation of knockout efficiency 5 days post electroporation with Cas9 in complex with purified sgRNA. As in panel **a**, edited organoids were seeded in matrigel or monolayer culture prior to lysis and western validation. Data representative of *n* = 2 independent experiments with similar results. RB1, cMYC, HSP90, and P53 were blotted on a single gel. EGFP, AR, and PTEN were blotted on a second gel with an independent HSP90 blot shown in Source Extended Data. **d**. Representative H&E stains (low and high magnification images) of established mouse models (Hi-MYC), human prostate cancer, and organoid OT-derived prostate tumors and are representative of *n* = 5 tumor samples. Data related to panels **a**-**c**. OT prostate tumors derived from transplantation of organoids grown in (top) monolayer or (bottom) traditional 3D matrigel conditions. **e**. Representative Synaptophysin (SYP) or ASCL1 IHC of tumors isolated from mice transplanted with RP organoids grown in (top) monolayer or (bottom) traditional 3D matrigel conditions. Data representative of *n* = 2 tumors per stain. **f**. Percentage of mice with tumors per genotype and organoid growth conditions. Sample size (mice) per genotype indicated within the figure panel. **g**. Survival of mice OT transplanted with 250k dissociated organoids grown in matrigel. Sample size (mice) per cohort indicated within the figure legend. **h**. Representative H&E stains of *n* = 1 mouse that developed OT tumors following transplantation of PtRM organoids grown in matrigel. **i**. Representative phospho-histone H3 IHC stains of PtPM or RPM prostate tumors isolated 4 weeks post OT. Histological classification performed using serial sectioned H&E. Dotted line represents the boundary of PRAD and NEPC. Images related to quantification shown in Fig. 1d. **j**. Average percentage of KRT8+ or KRT5+ tumor cells relative to total detected cells (DAPI+ nuclei) within RPM tumors 5 weeks post OT. Data represent the average positive cell number per tumor. Data derived from *n* = 5 RPM OT tumors. **k**. Average percentage of total ASCL1+ cells relative to total detected cells (DAPI+ nuclei) within RPM OT tumors at the indicated time points. Each data point represents the average marker positive cell per mouse tumor. 2–3 weeks, *n* = 4; 4–5 weeks, *n* = 6; 6–8 weeks, *n* = 8; and 10 weeks, *n* = 11 tumors. Statistics derived using one-way ANOVA with Tukey’s multiple comparisons correction. **l**. Percentage of cells staining positively for nuclear ASCL1 from tumors harvest 4 to 5 weeks post OT. Data represent the average positive cell number per tumor. Data derived from *n* = 4 (PtPM) and *n* = 5 (RPM) OT tumors per group. For panels **j**-**l**, error bars indicate mean and s.d. For panels **j**, **l** statistics derived using two-sided Student's *t*-test. All scale bars denoted within the figure panel. Source data

We next evaluated tumorigenicity following OT (Fig. 1a). Because expansion of organoids grown in 3D is labor intensive, we compared 3D expansion to short-term (5 day) monolayer expansion as a simpler alternative (Extended Data Fig. 2a–c). Although monolayer expansion was faster and generally yielded highly penetrant tumor growth (PtP, RP, PtPM and RPM), tumors invariably formed sarcomatoid-like histology uncharacteristic of human prostate tumors^22^ (Extended Data Fig. 2d–g). In contrast, tumors arising from organoids expanded exclusively in 3D culture consistently had histological phenotypes and lineage marker expression that remarkably mirrors human disease, particularly for the PtPM and RPM genotypes (Extended Data Fig. 2d–h). Phenotypes of each of the six combinations of genetic drivers, expanded using 3D or monolayer culture, are summarized in Supplementary Table 2. Due to the sarcomatoid-like histology seen following monolayer culture, all subsequent experiments used 3D expansion only. In summary, this faithful recapitulation of the complex histopathological features of advanced human prostate cancer represents a significant advance relative to traditional GEMMs and xenograft models.

### Rb1 loss is a critical gatekeeper event for NEPC transition

Having established highly penetrant PRAD using PtPM and RPM organoids, we comprehensively evaluated disease progression across both models (hereafter, PtPM and RPM mice). Histological assessment revealed that PtPM tumors consistently showed moderately to poorly differentiated PRAD histology during the first 2–3 weeks after transplantation. In contrast, RPM tumors displayed pockets of small cell-like nests with ‘salt-and-pepper’ chromatin and a mixture of trabecular or diffuse architecture suggestive of NEPC (Fig. 1b,c). The mitotic index in RPM tumors, particularly in NEPC regions (8–10 weeks) was greater than PtPM tumors, consistent with the rapid disease progression seen in patients with NEPC transformation (Fig. 1d and Extended Data Fig. 2i). Despite this difference in proliferation rate, the overall survival of PtPM mice was shorter, likely due to higher tumor engraftment potential as a 250-fold reduction in the number of injected PtPM cells results in comparable survival to RPM mice (Fig. 1e).

**Fig. 2:**
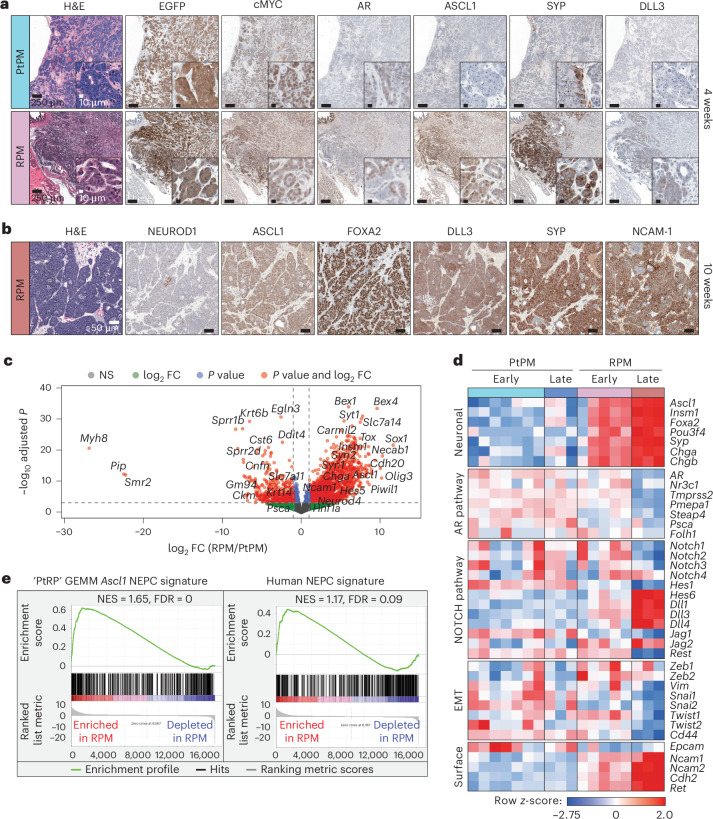
Molecular characterization of PtPM and RPM primary prostate tumor transplants demonstrates emergence of neuroendocrine carcinoma marker expression. **a**, Representative histological analysis of PtPM (top) and RPM (bottom) tumors isolated at 4 weeks after transplantation. Serial sections depict immunohistochemical staining of the indicated markers. Data are representative of *n* = 22 tumors. **b**, Representative histological analysis of RPM tumors isolated at 10 weeks after transplantation. Serial sections depict immunohistochemical staining of the indicated markers. Data are representative of *n* = 25 tumors. **c**, Volcano plot depiction of the log_2_ fold change in RNA expression of primary (OT) RPM tumors relative to primary (OT) PtPM tumors. Genes that meet or surpass the indicated thresholds of significance (two-sided Wald test with Benjamini–Hochberg multiple comparisons correction) and FC in expression are color coded as depicted in the figure legend. Data are derived from the comparison of PtPM (*n* = 10) and RPM (*n* = 8) tumors. **d**, Heatmap depicting the *z*-score normalized differential expression of select genes in PtPM versus RPM tumors. Time points of isolation are color coded in the figure as they are in **a**. Genes are grouped by the listed class or pathway. Early PtPM, 4 weeks; early RPM, ≤6 weeks. Late PtPM, 5 weeks; late RPM, 10 weeks. Data are related to samples used in **c**. **e**, Enrichment plots (GSEA) of established expression signatures of a GEMM of NEPC harboring conditional deletion of *Pten*, *Rb1* and *Trp53* (PtRP) (left) and histologically verified human NEPC within RPM primary tumors (right). FDR and normalized enrichment score (NES) are indicated in the figure. Analysis derived from the transcriptional profiles of multiple independent RPM tumors (*n* = 8) relative to PtPM tumors (*n* = 10). Data are related to samples used in **d**. All scale bars are noted in each panel and are of equivalent magnification across each marker. NS, not significant; FC, fold change. Source data

Early RPM tumors displayed more KRT8^+^ cells compared to KRT5^+^ cells, markers of luminal and basal identity, respectively (Extended Data Fig. 2j). Moreover, ASCL1 expression was observed by 4 weeks and increased by 8–10 weeks (Extended Data Fig. 2k). Regions with NEPC histology expressed canonical NE markers (FOXA2, DLL3, SYP and NCAM-1) and rarely NEUROD1 (refs. ^4,5,23^ and Fig. 2a,b). In contrast, tumors in PtPM mice contained rare ASCL1^+^ cells and never progressed to NEPC (Fig. 2a, Extended Data Fig. 2l and Supplementary Fig. 1). We conclude that functional *Rb1* loss is required for NEPC transformation, consistent with preclinical and clinical datasets demonstrating enrichment of *RB1* pathway mutations in small cell lung cancer (SCLC) and NEPC^23,24^.

**Extended Data Fig. 3:**
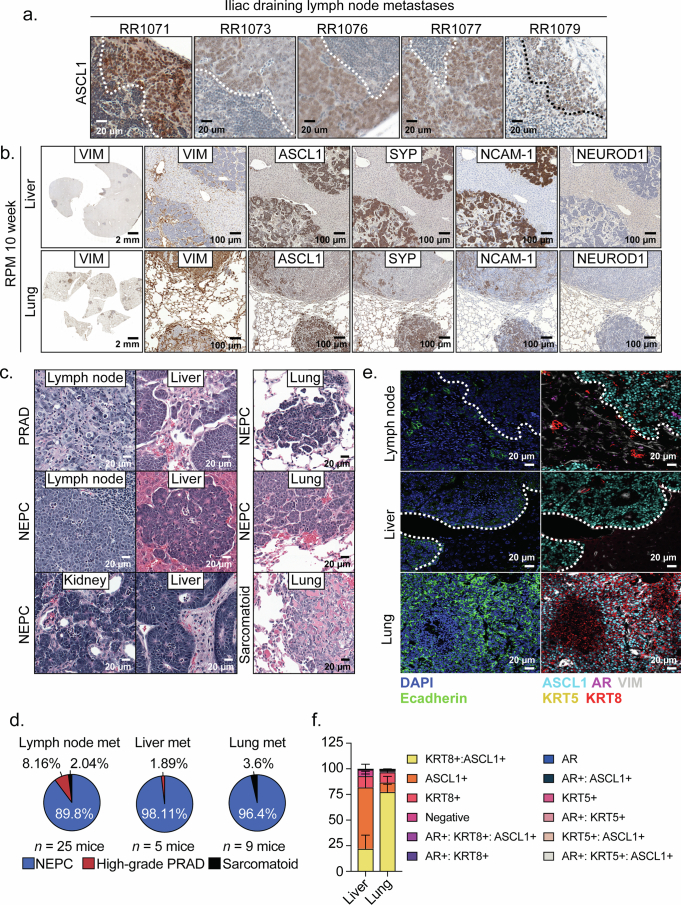
Metastatic tumors in RPM model establish with high penetrance NEPC histology and marker profile. **a**. Representative ASCL1 IHC stains from iliac lymph node metastases isolated from RPM OT mice. Dotted line represents the boundary of tumor and normal tissue. **b**. Representative serially sectioned immunohistochemical stains from metastases isolated across (top) liver and (bottom) lung tissue in RPM OT mice. Scale bars denoted in figure legend. VIM = VIMENTIN, SYP = SYNAPTOPHYSIN. **c**. Representative H&E stains and histological grade of metastases isolated from regional lymph nodes, kidney, liver, and lung tissue from RPM OT mice. Data related to panels **a**-**b**. Scale bars denoted within the figure panel. **d**. Pie charts demonstrating percentage of mice harboring distinct histotypes of prostate cancer in the indicated metastatic regions. Sample size denoted in figure panel. **e**. Representative mIF staining for lineage markers in metastastic tumors isolated from regional lymph nodes, liver, and lung tissue from RPM OT mice. Data representative of *n* = 3 mice. **f**. Percentage of unique cell types expressing the indicated lineage markers in RPM metastatic samples. For panels **a**-**d**, images and data are derived from *n* = 25 lymph node, *n* = 5 liver, and *n* = 9 lung metastatic samples from RPM OT mice. For panel **e**-**f**, images and data are representative of *n* = 3 lymph node, *n* = 3 liver, and *n* = 3 lung metastatic samples from RPM OT mice. Error bars denote mean and s.d. All scale bars and pseudo-coloring denoted in the figure panel. Source data

PtPM and RPM mice developed regional metastases in the draining iliac lymph nodes, but RPM mice also established distant metastases (primarily liver and lung; Fig. 1b,c,f), which retained the same NEPC profile observed in primary tumors, except for rare patches negative for ASCL1, SYP, NCAM-1 and NEUROD1 but occasionally positive for vimentin (VIM), a marker of mesenchymal-like cells (Extended Data Fig. 3a–d). Whether these ASCL1-negative regions reflect ongoing plasticity after metastasis of ASCL1^+^ cells, or independent metastatic events before NEPC transformation, requires further investigation. Notably, lung metastases in RPM mice contained a higher proportion of ASCL1^+^/KRT8^+^ cells compared to liver metastases. AR expression was absent in tumor cells at both metastatic sites (Extended Data Fig. 3e,f).

**Extended Data Fig. 4:**
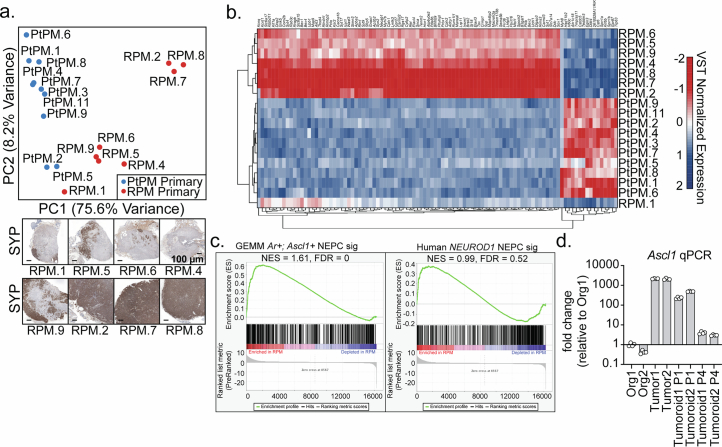
Bulk gene expression profiling of mouse tumors highlights the requirement for *Rb1* loss and the TME to induce and maintain NEPC fate. **a**. (Top) Principal component analysis of bulk RNA-seq data isolated from RPM or PtPM OT tumors. (Bottom) SYP immunohistochemical stains from the RPM tumors ordered by increasing percentage of SYP+ cells/tumor. RPM, *n* = 8 and PtPM, *n* = 10 tumors. Data related to Fig. 2a–d. **b**. Unsupervised hierarchical clustering of variant stabilized transcript normalized expression of the top 100 differentially expressed genes (columns) within tumors (rows). Data related Fig. 2c. **c**. GSEA enrichment plots of established expression signatures of (top) a GEMM of *AR* and *Ascl1*-co-expressing NEPC harboring conditional deletion of *Pten*, *Rb1*, and *Trp53* (PtRP), and (right) histologically verified human NEPC expressing *NEUROD1* within RPM primary tumors. FDR and NES indicated in the figure. Analysis derived from the transcriptional profiles of RPM tumors (*n* = 8) relative to PtPM tumors (*n* = 10). Data related to samples used in Fig. 2c, d. **d**. Quantitative PCR of *Ascl1* transcripts across *n* = 2 RPM organoids (org), tumors, and *n* = 2 tumor-derived organoids (tumoroids) at passage 1 (P1) or passage 4 (P4) post isolation. Each data point indicates technical quadruplicate values and bars represent mean and s.d. Data representative of *n* = 2 independent experiments with similar results. Source data

To further benchmark the PtPM and RPM models relative to autochthonous prostate cancer models and human samples, we performed bulk RNA sequencing of tumors collected early (PtPM ≤ 4 weeks and RPM ≤ 6 weeks) and late (PtPM = 5 weeks and RPM = 10 weeks). Consistent with the immunohistochemical findings, we observed progressive upregulation of genes involved in neuronal differentiation in RPM compared to PtPM tumors, including *Ascl1*, *Foxa2*, *Sox1*, *Chga* and *Olig3*, NOTCH pathway ligands and select transcription factors^4,25^ (for example, *Dll1*, *Dll3* and *Hes6*), as well as downregulation of AR and AR-target genes (for example, *Tmprss2*, *Pmepa1* and *Folh1*; Fig. 2c,d, Extended Data Fig. 4a,b and Supplementary Table 3). RPM tumors showed enrichment for NEPC transcriptional signatures from GEMMs and human specimens, demonstrating that RPM transplants recapitulate key molecular features observed in other preclinical models and clinical samples^16^ (Fig. 2e, Extended Data Fig. 4c and Supplementary Table 4). Further highlighting the critical role of the in vivo TME in initiating NEPC transformation, *Ascl1* transcript levels were ~2,000-fold higher in RPM tumors compared to long-term cultured RPM organoids. Moreover, the in vivo TME is required for maintenance of the NEPC state as *Ascl1* expression progressively declined in RPM tumors explanted to organoid culture (Extended Data Fig. 4d).

### The TME dynamically shifts throughout the NE transition

**Fig. 3:**
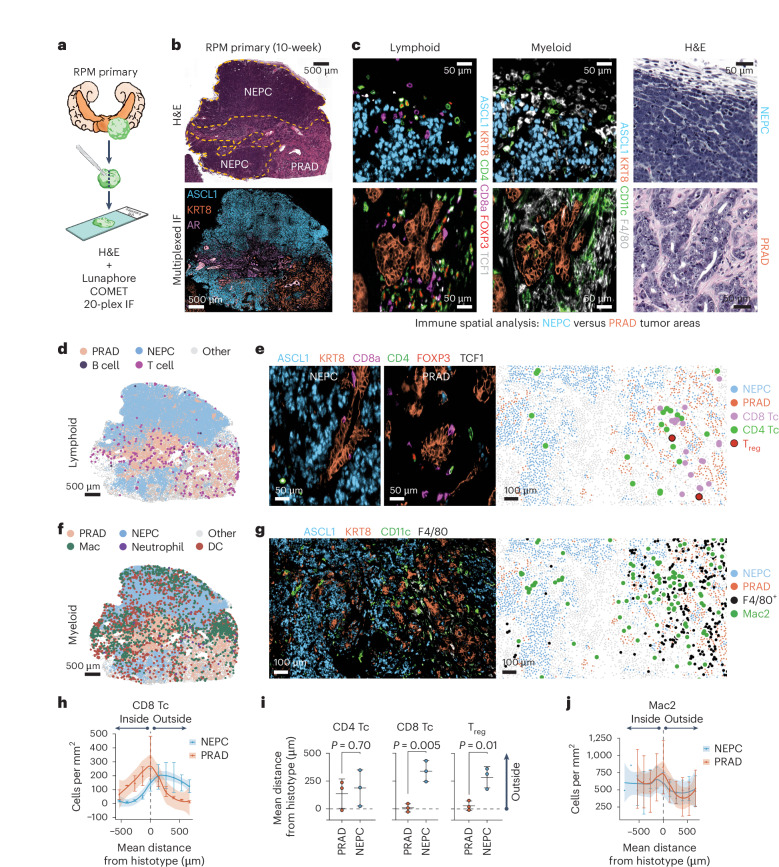
Multiplexed immunofluorescence identifies unique spatial distribution of immune cells within RPM prostate tumors, with local depletion of immune cell types in NEPC areas. **a**, Schematic representation of the methods used to process RPM tumors for 20-plex mIF. **b**, Representative H&E (top) and serial section (bottom) depicting a 3-marker pseudo-colored 10-week RPM tumor. Histological regions (PRAD versus NEPC) are denoted in the H&E and demarcated by dotted yellow line. **c**, Representative enhanced magnification of lymphoid (left) and myeloid cell markers (middle) and serially sectioned H&E (right). Staining was repeated independently twice with similar results. **d**, Representative segmented field of view (FoV) for the indicated general lymphoid cell types in a 10-week RPM tumor. **e**, Representative mIF of the indicated pseudo-colored lymphocyte markers within NEPC (left) or PRAD (middle). Data are presented as a segmented FoV indicating the localization of each lymphoid and tumor cell type in space (right). **f**, Representative segmented FoV for the indicated general myeloid cell types in a 10-week RPM tumor. **g**, Representative mIF of the indicated pseudo-colored myeloid and tumor histotype markers. Segmented FoV indicating the localization of each myeloid and tumor cell type in space (right). For **b**–**g**, images are representative of *n* = 3 tumors. For **d**–**g**, stains are representative of *n* = 2 experiments repeated with similar results. **h**, Frequency distribution of CD8^+^ T cells within binned distance outside or inside the defined interface region (NEPC or PRAD). **i**, Mean distance of the indicated cell types to the nearest histotype boundary. Statistics are derived from a two-sided Student’s *t*-test. Error bars denote mean and s.d. **j**, Frequency distribution of Mac2 cells (CD11b^lo^; CD11c^+^; F4/80^+^) within each binned distance outside or inside of the defined interface region (NEPC or PRAD). Data are calculated as in **h**. For **h**,**j** error bar represents mean and s.e.m. of the cell counts per bin. Shaded regions in **h**,**j** were approximated through the Loess method. The dotted line in **h**–**j** represents the boundary of the tumor histotype or tumor edge. Scale bars are denoted within each panel. Data derived from *n* = 3 independent tumor samples. Infiltration analyses are representative of *n* = 3 distinct NEPC and PRAD boundaries. Source data

**Extended Data Fig. 5:**
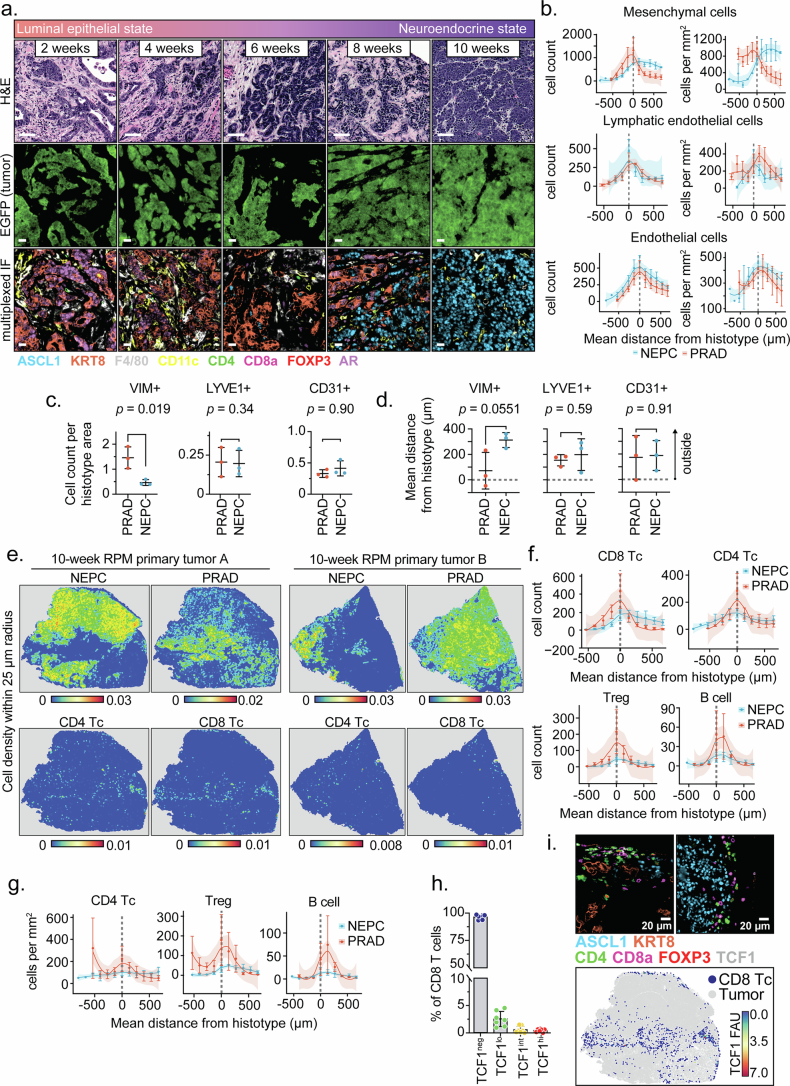
RPM tumors dynamically transition into NEPC and establish strong T cell exclusion. **a**. Representative (top) H&E, (middle) EGFP, and mIF (bottom) of RPM tumors isolated at the indicated time points. EGFP and mIF images are matched sections. Data representative of *n* = 3 tumors stained by COMET. Scale bar for all images indicates 20 μm. Pseudo-coloring listed within the figure panel. **b**. Cell number of each indicated stromal and vascular cell types within each binned distance outside or inside the defined interface region (NEPC or PRAD). **c**. Frequency distribution of each indicated stromal and vascular cell types within each binned distance outside or inside the defined interface region (NEPC or PRAD). **d**. Dot plot depicting mean cell density outside or inside NEPC or PRAD tumor regions for the indicated non-immune stromal cell types. VIM+, mesenchymal cells: LYVE1+, lymphatic endothelial cells; CD31+, endothelial cells. Statistics for panels **c**-**d** derived from two-sided Student's *t*-test. **e**. Spatial cell type density (cell density within 25 μm radius) for the indicated tumor cell types and lymphocytes across 10-week RPM tumors (*n* = 2). Data representative of *n* = 3 RPM tumors with similar results. Heatmap represents average cell density (cell number/μm^2^). **f**. Mean lymphocyte cell count relative to nearest PRAD or NEPC boundary. **g**. Data as in panel **f** but normalized to the binned tumor area. **h**. Percentage of TCF1-negative (neg), intermediate (int), or high (hi) CD8 T cells within RPM tumors. Data points represent the mean number of indicated CD8 T cells across TCF1 expression groups and error bars denote standard deviation. **i**. (Top) Representative mIF stains from RPM 10-week tumors of the indicated lymphocyte markers. (Bottom) Segmented FoV where each dot represents a CD8 T cell coordinates within a 10-week RPM tumor section. Each dot is color coded based on predetermined thresholds for TCF1 expression, FAU = fluorescence arbitrary units. Data related to panel **h**. Dotted line in panels **b**, **d**, **f**-**g** represents the boundary of the histotype to a different histotype or the edge of a tumor. Positive values indicate cells found outside the histotype boundary; negative values indicate cells found inside the histotype boundary. Data and images in panels **h**-**i** derived from *n* = 7 tumors. Data in panels **b**-**d**, **f**-**g** derived from *n* = 3 tumors. Error bars denote mean and s.e.m. in panels **b**, **f**, and **g** and mean and s.d. in panels **c**-**d**, **h**. Dotted line in panels **b**, **d**, **f**-**g** indicate the boundary of a PRAD or NEPC tumor region. Smoothened data curves in panels **b**, **f**, and **g** fit by Loess method. Source data

Given the requirement of the in vivo setting for lineage plasticity, we developed a 20-marker multiplexed immunofluorescence (mIF) panel to visualize prostate tumor cells in the context of adjacent immune populations, vasculature and stroma throughout tumor progression (Fig. 3a–g and Supplementary Tables 5 and 6). We focused on the later stages of tumor progression within the RPM model to identify changes in the TME within large NEPC regions (Fig. 3b). We used GFP, together with KRT8 and AR or ASCL1 coexpression to distinguish PRAD from NEPC (Methods and Extended Data Fig. 5a). After mapping these regions across multiple tissue sections from RPM tumors containing NEPC patches, we looked for selective changes in cell type composition within the TME.

Focusing initially on stroma, we noted that mesenchymal cells were abundant in PRAD but depleted in NEPC regions. We observed a similar trend for LYVE1^+^ lymphatics although this did not reach statistical significance. CD31^+^ endothelial populations remained unchanged (Extended Data Fig. 5b–d).

Turning to immune cells, we noted striking depletion of CD8^+^ and FOXP3^+^;CD4^+^ regulatory T (T_reg_) cells as well as F4/80^+^ macrophages in NEPC regions, consistent with reports of immune exclusion within human NE cancers^24,26,27^. Conversely, FOXP3^−^;CD4^+^ T cells were equally distributed within PRAD and NEPC, with many located at PRAD boundaries, suggestive of differential recruitment and retention of T cell subsets between histologies (Fig. 3d,e,h,i and Extended Data Fig. 5e–g). Most (~96%) CD8^+^ T cells in PRAD regions were TCF1-negative, aligning with previous findings demonstrating downregulation of TCF1 and upregulation of effector programs in tumor infiltrating compared to draining lymph node resident CD8^+^ T cells^28^ (Extended Data Fig. 5h,i).

**Extended Data Fig. 6:**
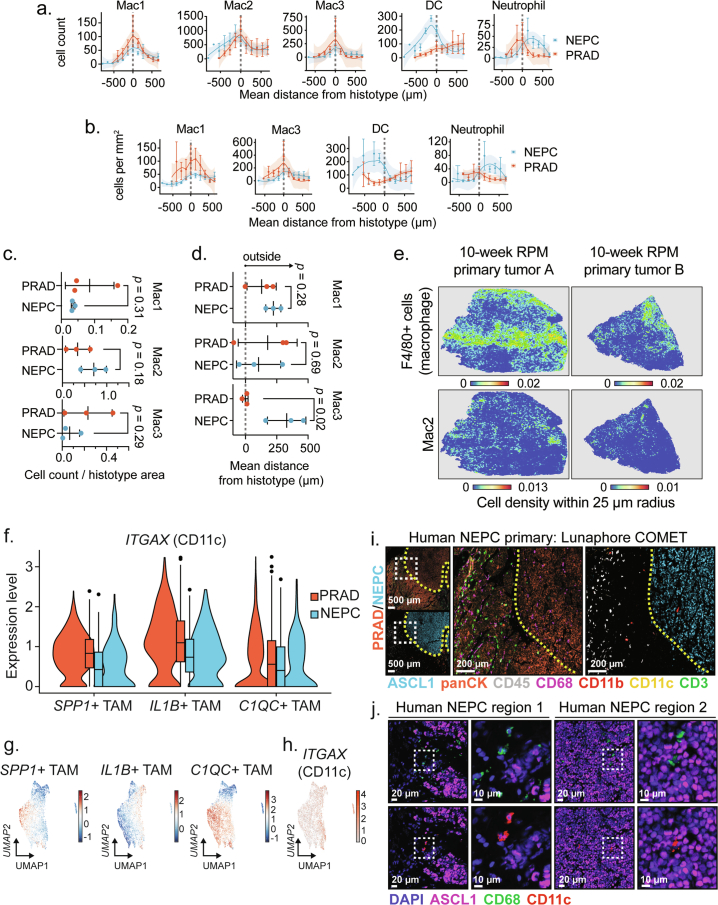
A subpopulation of tumor-associated macrophages is retained in mouse and human NEPC. **a**. Frequency distribution of each indicated cell type within each binned distance outside or inside the defined interface region (NEPC or PRAD). **b**. Frequency distribution of each indicated cell type density within each binned distance from defined interface region (NEPC or PRAD). **c**. Dot plot depicting mean cell density away from the boundary of NEPC or PRAD tumor regions for the indicated macrophage subtypes. **d**. Dot plot depicting mean cell distance (μm) away from the boundary of NEPC or PRAD tumor regions for the indicated macrophage subtypes. **e**. Spatial cell type density (cell density within 25-μm radius) for the indicated myeloid cell types across 10-week RPM tumors (*n* = 2). Heatmap represents average cell density (cell number/μm^2^) and are representative of *n* = 3 RPM tumors with similar results. **f**. *ITGAX* (CD11c) expression across previously published tumor-associated macrophage populations identified within human PRAD (*n* = 9) and NEPC (*n* = 3) tumors sequenced by scRNA-seq. Violin and box plots are median centered and depict the 25th and 75th percentiles of *ITGAX* expression, respectively. **g**. Gene expression modules of TAM subsets identified within human PRAD and NEPC samples displayed in UMAP space, related to panel **f**. Scale bar represents module score. **h**. *ITGAX* (CD11c) expression across all myeloid cell types identified within human PRAD and NEPC samples displayed in UMAP space, related to panels **f**-**g**. Scale bar represents raw expression counts. **i**. Representative mIF of a human prostatectomy verified to contain mixed PRAD and NEPC pathology. Left zoomed out panels contain white dotted line indicating zoomed in regions on the right. Dotted yellow line represents the boundary of a pan-cytokeratin+ (panCK) PRAD and ASCL1+ NEPC. For panels **h**-**i**, images are representative of *n* = 1 human mixed histology tumor sample imaged across multiple distinct tumor regions of interest. Staining was repeated *n* = 2 times with similar results. **j**. Representative mIF of two distinct regions within the human NEPC sample shown in panel **i**. Dotted square indicates magnified inset shown adjacent to lower magnification view. Error bars in panels **a**-**b** represent mean and s.e.m. across *n* = 3 RPM tumors. Error bars in panels **c**-**d** represent mean and s.d. across *n* = 3 RPM tumors. Statistics in panel **c**-**d** derived by two-sided Student's *t*-test. Dotted line in panels **a**-**b**, **d** represents the boundary of the histotype to a different histotype or the edge of a tumor. Positive values indicate cells found outside the histotype boundary; negative values indicate cells found inside the histotype boundary. Smoothened data curves in panels **a**-**b** fit by Loess method. Source data

We identified five distinct myeloid populations, which we labeled Mac1 (CD11b^+^;F4/80^−^), Mac2 (CD11b^lo^;CD11c^+^;F4/80^+^), Mac3 (CD11b^+^;F4/80^+^), neutrophil (CD11b^+^;Ly6G^+^;S100A9^+^) and dendritic cells (DCs; CD11c^+^;F4/80^−^; Extended Data Fig. 6a and Supplementary Table 6). Neutrophil infiltration was confined to PRAD boundaries (Fig. 3f and Extended Data Fig. 6a,b). Mac1 and Mac3 populations were largely absent in NEPC; however, Mac2, which harbors similar marker expression as alveolar and wound-healing macrophages was present within NEPC^29^ (Fig. 3f,g,j and Extended Data Fig. 6c–e). We were surprised to also see many CD11c^+^;F4/80^−^ cells within NEPC regions of primary tumors, raising the possibility of DC infiltration (Fig. 3f and Extended Data Fig. 6a,b).

To determine whether the differences in PRAD versus NEPC immune infiltrates in RPM mice are seen in human prostate cancer, we examined a published human single-cell RNA sequencing dataset that includes PRAD and NEPC samples^16^. Both histologies had myeloid infiltration, but NEPC harbored fewer tumor-associated macrophages (TAMs) relative to PRAD (Extended Data Fig. 6f and Methods). CD11c (*ITGAX*) expression was evident across TAM populations within both PRAD and NEPC, and highest in *IL1B*^+^ TAMs (Extended Data Fig. 6f–h). A decrease in immune infiltration was also observed in NEPC regions of a prostatectomy specimen from a patient with mixed PRAD/NEPC histology, with CD11c^+^;CD68^+^ TAMs present within ASCL1^+^ tumor areas (Extended Data Fig. 6i,j). Whether these CD11c^+^ myeloid populations correspond to professional antigen-presenting cells requires further phenotypic (for example, MHC-II, CD103 and BATF3 expression) and functional characterization. Nonetheless, early CD8^+^ T cell infiltration in PRAD and persistence of potential DCs in late-stage NEPC in this model suggest that deeper analysis may be informative in addressing the disappointing clinical results seen with conventional immune checkpoint blockade therapy in prostate cancer.

**Fig. 4:**
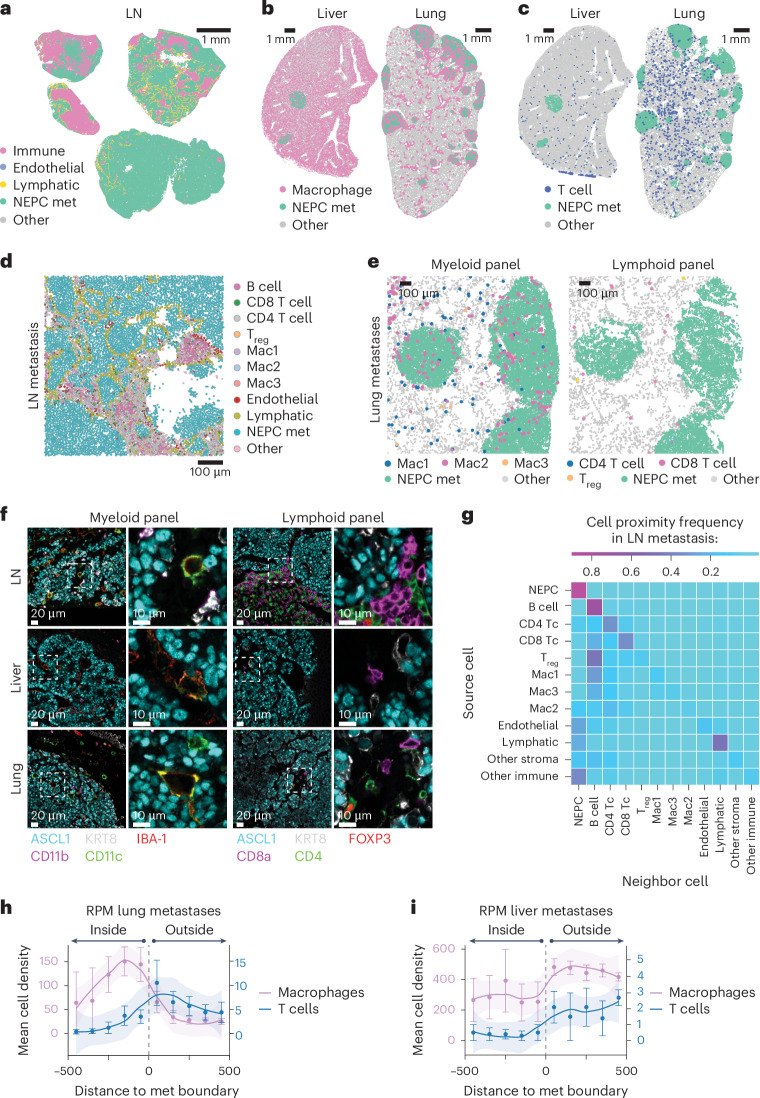
NEPC metastatic lesions are T cell excluded but retain macrophage infiltrates. **a**, Representative segmented FoV for the indicated cell types within *n* = 4 draining lymph node (LN) metastases derived from *n* = 2 mice (OT) with RPM tumors. **b**, Representative segmented FoV of macrophages (IBA-1^+^) within liver or lung sections (*n* = 3 mice each) obtained from mice (OT) with RPM tumors. Note, liver-resident macrophages (Kupffer cells) are IBA-1^+^. **c**, Representative segmented FoV of T cells (CD4^+^ or CD8^+^) within liver or lung sections (*n* = 3 mice each) obtained from mice (OT) with RPM tumors. **d**, Representative zoomed in segmented FoV for all cell types listed within a draining LN metastasis. Scale denotes relative cell size. Image representative of *n* = 4 lymph nodes with similar results. **e**, Representative zoomed in segmented FoV across serial lung sections obtained from mice (OT) with RPM tumors, identifying NEPC metastatic nodules infiltrated with (left) macrophage subsets or (right) T cell subsets. Images representative of *n* = 3 lung sections stained for myeloid or lymphoid panel with similar results. **f**, Representative mIF of the indicated cell type markers across distinct metastatic sites obtained from mice OT transplanted with RPM organoids. Images are representative of *n* = 3 mouse samples for indicated each tissue. Staining was repeated independently twice with similar results. **g**, Neighborhood composition heatmap of cell types found within RPM draining LN metastases demonstrating the proximity of the source cell relative to a neighboring cell (20-pixel distance). Data are derived from *n* = 4 independent metastatic LN samples isolated from *n* = 2 mice. **h**, Frequency distribution for macrophages (IBA1^+^) or T cells (CD4^+^ or CD8^+^) within each binned distance outside or inside of RPM lung metastatic samples. **i**, Frequency distribution for macrophages (IBA1^+^) or T cells (CD4^+^ or CD8^+^) within each binned distance outside or inside of RPM liver metastatic samples quantified as in **h**. Shaded region in panels **h**,**i** approximated through the Loess method. Error bar in **h**,**i** represents mean and s.e.m. of the cell counts per bin. Dotted line in **h**,**i** represents the boundary of a tumor histotype or tumor edge. All metastatic tumors per section within an individual mouse were combined for infiltration analysis and subsequently averaged between replicates (*n* = 3 mice). Source data

We next profiled RPM metastases, a clinically relevant site of NEPC histology (Fig. 4a–c). Lymph node metastases showed prominent exclusion of CD45^+^ cells within ASCL1^+^ tumor nests, highlighting the capacity of NEPC to promote immune exclusion within lymphocyte-dense microenvironments (Fig. 4a,d). Distant metastases (liver and lung) showed exclusion of T_reg_, CD4^+^ and CD8^+^ T cells, but retention of IBA-1^+^ macrophages that co-stain with markers consistent with Mac1, Mac2 or Mac3 identities, a finding confirmed by neighborhood composition analysis (Fig. 4b,c,e–i and Methods). Taken together, primary tumors and metastases displayed T cell exclusion in NEPC but retained subsets of myeloid cells such as Mac2 and those with DC-like cell surface marker expression (CD11c^+^F4/80^−^). Critically, our syngeneic models are suitable for studies using model antigens to evaluate strategies to overcome the immunosuppressive prostate TME.

### NEPC arises from tumor cells with luminal features

**Fig. 5:**
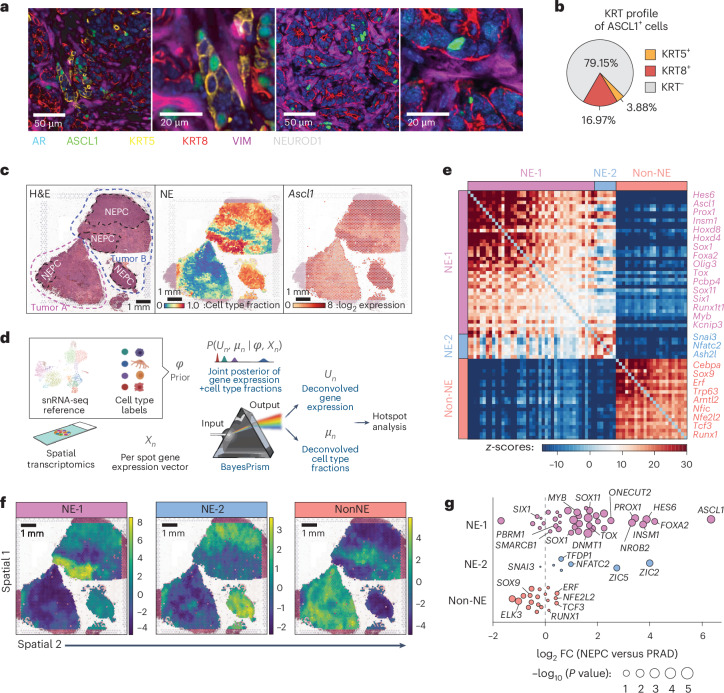
PrismSpot reveals spatial transcriptomic heterogeneity within NEPC marked by *Ascl1* coexpressed with distinct NE-related TFs. **a**, Representative confocal images of 7-plex mIF of the indicated markers. Second and fourth images are digitally magnified versions of the first and third panel from the left. Data are representative of *n* = 29 RPM tumors at varying time points post OT. **b**, Percentage of all ASCL1^+^ cells coexpressing KRT5, KRT8 or KRT-negative within an individual RPM OT tumor. Data are derived from the average percentage of cells within each tumor across *n* = 10 tumors 6 weeks after OT. **c**, H&E stains of 10-week RPM tumors (*n* = 2) (left). Tumors A and B are outlined in red and blue dotted lines, respectively. NEPC regions are highlighted in black dotted lines. BayesPrism inferred cell type fraction for NEPC (middle). The log_2_ fold expression of *Ascl1* overlayed on the tumor histology (right). **d**, Workflow of PrismSpot method. BayesPrism infers the posterior of cell-type-specific gene expression (U) and cell type fraction (μ) of each spot. The expression profile of the cell type of interest (NEPC) was selected as the input for Hotspot analysis. **e**, Heatmap shows PrismSpot output of the pairwise local correlation *z*-scores of *n* = 71 TFs of high consensus scores (>0.8) and significant spatial autocorrelation (FDR < 0.01). TFs are clustered into *n* = 3 modules based on pairwise local correlations between all TFs of significant spatial autocorrelation. **f**, Spatial expression patterns of TFs within each module are visualized using smoothed summary module scores. Images are representative of *n* = 2 RPM OT tumors. Spatial analyses were repeated on technical replicates with similar results across *n* = 2 tumors. **g**, Beeswarm plot shows the log_2_ FC in expression of TFs in each module between bulk RNA-seq of human NEPC (*n* = 9) and PRAD (*n* = 50) samples. Dot size shows the two-sided *P* values based on a Wilcoxon test. All scale bars are indicated within each figure panel.

The dynamic nature of the RPM model also allows for careful examination of the earliest stages of NEPC transformation. ASCL1, a marker of emerging NE cells, was detected at 4–6 weeks, appearing as EGFP^+^;KRT8^+^;ASCL1^+^ tumor cell clusters (Fig. 5a and Extended Data Figs. 2k and 5a). By 10 weeks, larger homogeneous clusters of ASCL1^+^;KRT8^−^ tumor cells with NEPC histology were visible. The observation that the earliest detectable ASCL1^+^ cells coexpress KRT8 suggests that NE cells may arise from KRT8^+^ luminal cells. Indeed, KRT8^+^;ASCL1^+^ cells were 4–5-fold more abundant than KRT5^+^;ASCL1^+^ cells at 6 weeks (*P* = 0.025, two-tailed Student’s *t*-test, Fig. 5b). These findings are consistent with previous human basal prostate organoid transplantation studies^30,31^, although our murine platform has the advantage of culturing and transforming both basal and luminal subsets in vitro before expansion of a luminal tumor population in vivo (Extended Data Figs. 1e and 2j). By 8–10 weeks, primary and metastatic tumor cells were mostly AR^−^ and ASCL1^+^ with heterogenous KRT8 and E-cadherin expression (Figs. 3b and 5b and Extended Data Figs. 3e,f and 5a).

The appearance of histologically homogeneous, spatially separate clusters of highly proliferative NE cells within weeks of detecting isolated ASCL1^+^;KRT8^+^ cells is consistent with rapid clonal expansion. To characterize the degree of tumor heterogeneity, we performed spatial transcriptomics (10x Visium) and single-cell nuclear RNA sequencing (snRNA-seq) from 10-week RPM tumors containing mixed PRAD-NEPC histology (Fig. 5c). We observed distinct NE tumor cell clusters from snRNA-seq with variable KRT8 expression (Supplementary Fig. 2a,b), consistent with the heterogeneity observed in NEPC by mIF.

**Extended Data Fig. 7:**
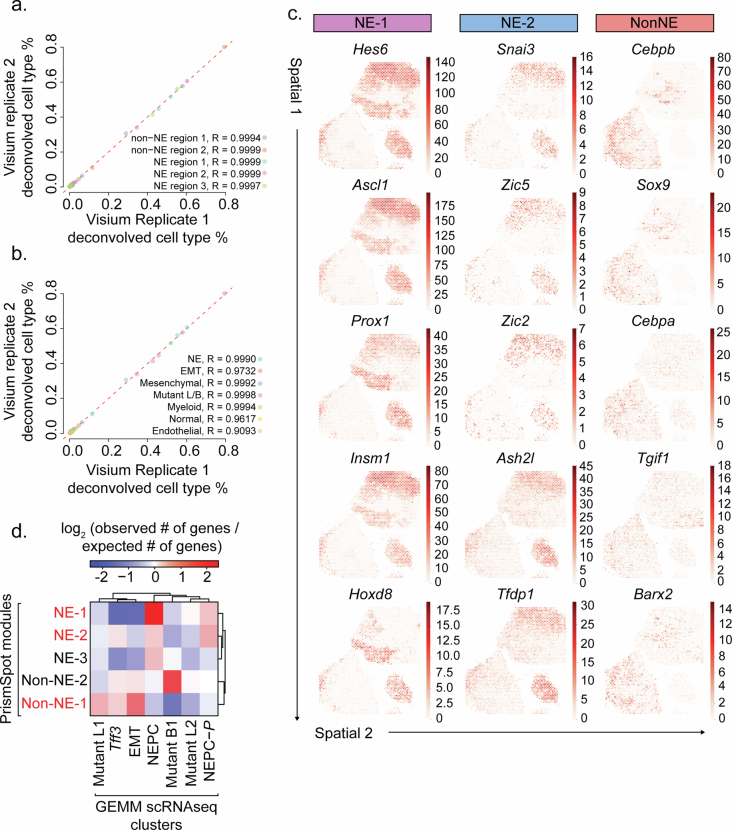
RPM tumors establish NEPC with heterogeneous expression of distinct NE TFs. **a**. Scatter plot shows the mean cell type fraction inferred by BayesPrism across *n* = 2 technical replicates for each cell type in each region, colored by regions defined by histology. **b**. Same as panel **a**. but colored by cell type. NE = neuroendocrine, EMT = epithelial-to-mesenchymal transition tumor cells, Tumor L/B = tumor cells with gene expression associated with luminal and basal lineages. **c**. Spatial expression (10X Visium) across the indicated RPM tumor (*n* = 2) spatial modules identified by PrismSpot (related to Fig. 5e, f). Scale bar represents raw expression counts. **d**. Heatmap of the observed gene overlap normalized to expected gene overlap between PrismSpot modules and several tumor clusters derived by previously published GEMM models of NEPC. Robust PrismSpot modules identified in red font.

Given the mixture of multiple cell types within individual tissue spots used for spatial transcriptomic sequencing, we applied BayesPrism^32,33^ to deconvolve tumor cell from nontumor cell transcripts using snRNA-seq data as the reference (Fig. 5d). BayesPrism integrates a single-cell genomics reference with spatial transcriptomics data to deconvolve each spot into the cell type fractions present and provide a cell-type-specific count matrix for each spot^32,33^. Before deploying BayesPrism for further downstream analysis, we assessed the robustness of the inferred deconvolution by comparing BayesPrism on technical replicates profiled from adjacent tissues and found strong correspondence of inferred cell type fraction (Extended Data Fig. 7a,b), for example, recapitulation of NEPC distribution observed by histology (Fig. 5c).

We next investigated the expression of transcription factors (TFs) within histologically confirmed and BayesPrism inferred NEPC regions. All NEPC regions showed *Ascl1* expression with minimal *Neurod1* and *Pou2f3* expression (Supplementary Fig. 3). Conversely, other TFs previously implicated in NEPC (for example *Mycn*, *Onecut2*, *Pou3f2* and *Pou3f4*) and cerebellar development (*Olig3*) were expressed in subsets of NEPC regions^12,34–37^ (Supplementary Fig. 3). The spatial heterogeneity in expression of these selected TFs, as well as similar TF heterogeneity reported in SCLC^38–41^ led us to examine the structure underlying this heterogeneity using Hotspot^42^, which identifies patterns in spatially-varying genes. The limited resolution of Visium technology makes identification of gene modules associated with a single cell type of interest challenging due to colocalization of genes expressed within multiple cell types or between a pair of colocalized cell types. To overcome this, we leveraged a powerful feature of BayesPrism: inference of cell-type-specific count matrices, thereby associating each transcript with its respective cell type (Methods). As input to Hotspot, we used the deconvolved tumor count matrices, a strategy we term ‘PrismSpot’, a combination of BayesPrism and Hotspot (Fig. 5d). Compared to directly applying Hotspot on un-deconvolved Visium data, the spatial auto- and pairwise correlation computed by PrismSpot showed significantly stronger signal-to-noise for tumor-specific gene modules (Supplementary Fig. 4a–g) over marker genes derived from a GEMM scRNA-seq dataset (Supplementary Fig. 5a–c). Upon iterative subsampling of the Visium data (Methods), we narrowed our gene list to 71 TFs defining two NEPC states (NE-1 and NE-2) and a single PRAD state (Non-NE; Fig. 5e,f and Supplementary Table 7).

NE-1, whose leading genes include coordinated expression of *Ascl1* and other TFs implicated in neuronal biology^43^ (*Hes6*, *Prox1* and *Insm1*), was enriched across all NEPC regions and corresponded to regions with high *Mycn* and *Olig3* expression as well as KRT8^+^;ASCL1^+^ tumor cells (Fig. 3b and Supplementary Fig. 3). NE-2, defined primarily by *Nfatc2* (a regulator of *Tox* expression within lymphocytes^44–46^) and the epithelial-to-mesenchymal (EMT) TF *Snai3* was enriched in some but not all NEPC regions (Fig. 5f and Extended Data Fig. 7c). *Nfatc2* expression has been linked with an EMT-like state in melanoma^47^. Both NE spatial modules were selectively enriched in a previously reported NEPC GEMM signature^16^ (Extended Data Fig. 7d) as well as human NEPC^48^ (NE-1, *P* = 1.17 × 10^−7^; NE-2, *P* = 5.50 × 10^−4^; Non-NE, *P* = 0.742, two-sided Wilcoxon test; Fig. 5g and Supplementary Table 8). Collectively, mIF and spatial transcriptomics implicate KRT8^+^ luminal epithelial cells as the source of NEPC, which evolves into spatially distinct ASCL1^+^ subpopulations with heterogeneous expression of NE-associated TFs.

### Ascl1 is essential for NEPC transformation

**Fig. 6:**
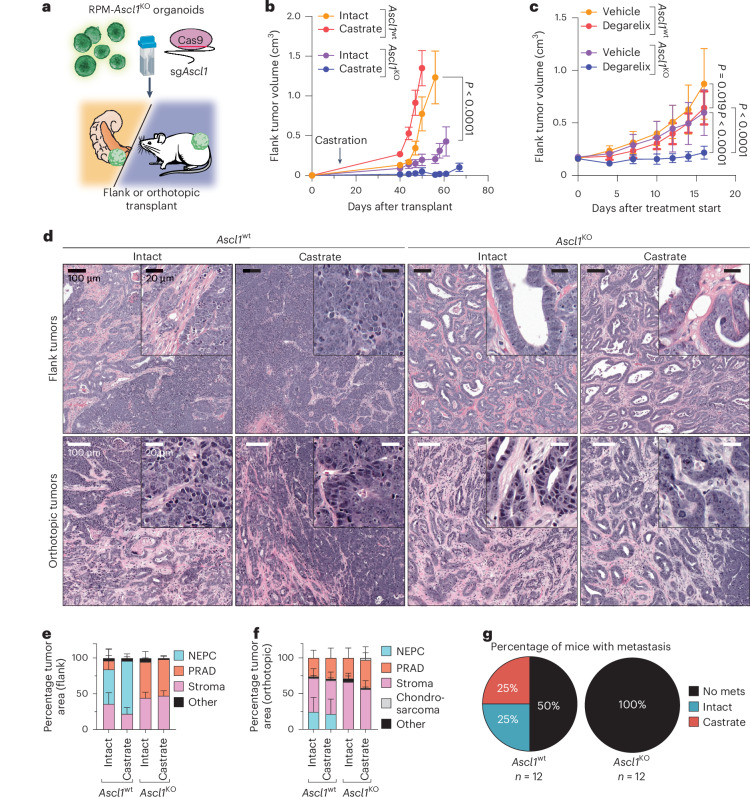
Loss of *Ascl1* results in abrogated NEPC establishment and castration-sensitivity. **a**, Schematic for the generation of RPM-*Ascl1*^wt^ and RPM-*Ascl1*^KO^ tumors transplanted into the flanks or prostates of immunocompetent C57BL/6J hosts. **b**, Longitudinal SQ tumor volumes of the indicated tumor genotypes and host backgrounds. Statistics derived using two-way ANOVA with Tukey’s multiple comparisons correction for data collected between days 0–56 to ensure equal sample size comparisons. Error bars denote mean and s.e.m. *n* = 6 tumors across each group. Castration or sham surgery performed 14 days post SQ transplantation. **c**, Longitudinal SQ tumor volumes of the indicated tumor genotypes and host backgrounds. Statistics were derived using two-way ANOVA with Tukey’s multiple comparisons correction for data collected between 0–16 days post treatment start to ensure equal sample size comparisons. Error bars denote mean and s.d. *Ascl1*^wt^-vehicle, *Ascl1*^wt^-vehicle and *Ascl1*^KO^-degarelix, *n* = 8 tumors; *Ascl1*^KO^-vehicle, *n* = 9 tumors. Vehicle or degarelix treatment was initiated upon tumor establishment (≥150 mm^3^). **d**, Representative H&E of SQ (top) and OT (bottom) tumors isolated at end point. Genotype and treatment groups are listed within the figure panel. Scale bars are denoted within the figure panel. Data are related to samples in **b**,**c** and Extended Data Fig. 8c,d. **e**, Stacked bar charts representing percentage of OT tumor area composed of the histological categories depicted in the figure legend. Data are quantified tumor histology compared in **b** and represent average tumor area. **f**, Stacked bar charts representing percentage of SQ tumor area composed of the histological categories depicted in the figure legend. Data are quantified tumor histology compared in **c** and represent the average tumor area. For **e**,**f**, error bars indicate mean and s.d. **g**, Pie charts representing percentage of mice with metastatic disease (regional and distal) in intact or castrated hosts of the indicated genotypes. Statistics are derived from a two-sided Fisher’s exact test, *P* = 0.0137. The number of biological replicates is indicated in the figure panel. Scale bars are denoted in the figure panels. Source data

**Extended Data Fig. 8:**
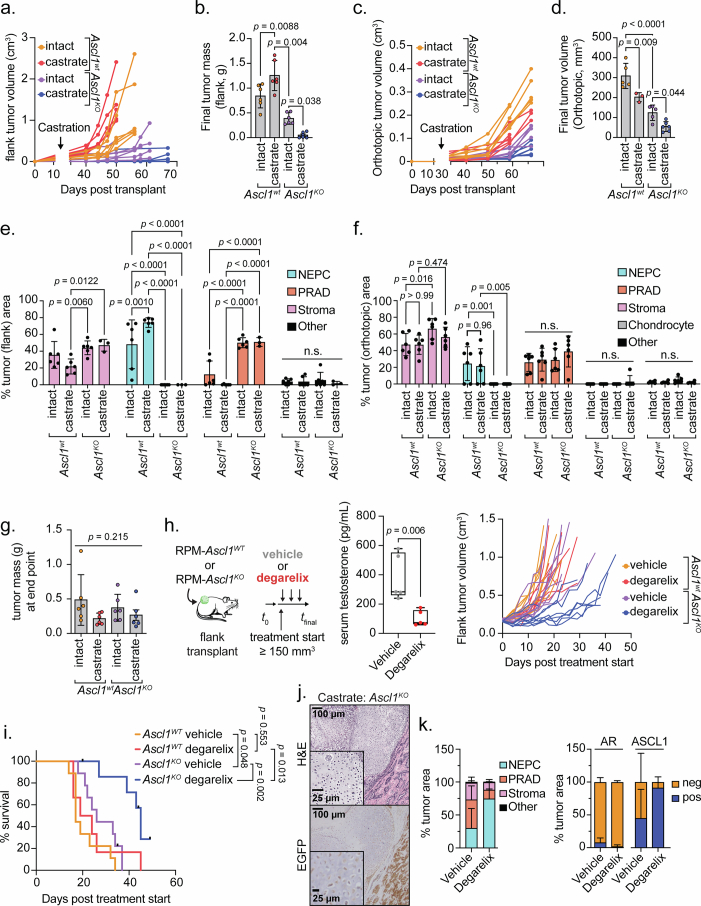
*Ascl1* loss prior to NE transition confers androgen dependence. **a**. Individual longitudinal SQ tumor volumes as determined by caliper. Castration or sham surgery was performed 14 days post organoid transplantation. *n* = 6 tumors across each group. Data related to Fig. 6b. **b**. Final SQ tumor mass at experimental end point. *n* = 6 tumors across each group. Statistics derived by one-way ANOVA with Sidak’s multiple comparisons correction. **c**. Individual longitudinal OT tumor volumes determined by ultrasound. *n* = 6 tumors per group. Castration or sham surgery was performed 14 days post organoid transplantation. **d**. Final OT prostate tumor volumes determined by ultrasound. Measurements derived from *Ascl1*^*wt*^ intact *n* = 5; *Ascl1*^*wt*^ castrate *n* = 3; *Ascl1*^*KO*^ intact *n* = 5; and *Ascl1*^*KO*^ castrate *n* = 6 mice. Statistics derived by one-way ANOVA with Sidak’s multiple comparisons correction. **e**. Bar charts representing percentage of SQ tumor area composed of the histological categories depicted in the figure legend. Data related to Fig. 6e. Each dot represents the average area per tumor. Statistics derived by two-way ANOVA with Tukey’s multiple comparisons correction. Error bar denotes mean and standard deviation. *n* = 6 tumors per group, except for the *Ascl1*^*KO*^ castrate group (*n* = 3 tumors). **f**. Bar charts representing percentage of OT tumor area composed of the histological categories depicted in the figure legend. Data related to Fig. 6f. Each dot represents the average area per tumor. Statistics derived by two-way ANOVA with Tukey’s multiple comparisons correction. Error bar denotes mean and s.d. *n* = 6 tumors per group. **g**. Final OT tumor mass at experimental end point. *n* = 6 tumors per group. Statistics derived by one-way ANOVA. Data related to panel **c**; however, after cessation of ultrasound measurements, tumors were isolated and weighed once palpable or distress was observed. **h**. (Left) schematic of SQ transplantation assay by which mice are randomized into vehicle or degarelix treatment arms after tumors reach predetermined volume as measured by caliper. (Middle) Validation of serum testosterone depletion assessed by ELISA collected 2 weeks post single dose of degarelix (15 mg/kg) or vehicle (5% mannitol). Box and whisker plot denotes minima and maxima data points, are mean centered, and display the top (90th) and bottom (10th) percentiles. Statistics derived using two-sided Student’s *t*-test. Data derived from serum measurements isolated from vehicle, *n* = 5; and degarelix, *n* = 5 mice. (Right) Longitudinal SQ caliper measurements of RPM-*Ascl1*^*WT*^ vehicle and degarelix treated *n* = 8, RPM-*Ascl1*^*KO*^ vehicle *n* = 9, RPM-*Ascl1*^*KO*^ degarelix *n* = 8 mice per group. **i**. Survival of vehicle or degarelix treated mice with SQ transplants of the indicated RPM organoid genotypes. Statistics derived from the Log-rank (Mantel-Cox) test for each pairwise comparison. Data related to Fig. 6c and panel **h**. RPM-*Ascl1*^*WT*^ vehicle *n* = 9, RPM-*Ascl1*^*WT*^ degarelix *n* = 6, RPM-*Ascl1*^*KO*^ vehicle *n* = 9, and RPM-*Ascl1*^*KO*^ degarelix *n* = 8 mice. **j**. Representative (left) H&E stain and (right) IHC of EGFP of RPM-*Ascl1*^*KO*^ tumor (*n* = 1) growth in a castrated host demonstrating chondrosarcomatoid histopathology. Scale bars denoted within the figure. **k**. (Left) Stacked bar charts representing percentage of SQ tumor area composed of the histological categories depicted in the figure legend. Data are quantified histology of RPM tumors treated with vehicle (*n* = 5 mice) or degarelix (*n* = 4 mice) for 4 weeks after tumor establishment (≥150 mm^3^) and represent average tumor area. (Right) Stacked bar charts of the percentage of AR- and ASCL1-pos (positive) or neg (negative) tumor cells (defined as EGFP+; CD45-; VIMENTIN-) within vehicle or degarelix treated RPM SQ tumors. Vehicle *n* = 5; and Degarelix *n* = 4 tumors. Error bars in panels **b**, **d**, **e**-**f**, **g**, and **k** indicate mean and s.d. Source data

In addition to its role as a master TF in neural lineage specification^49,50^, several human SCLC cell lines and at least one human NEPC xenograft model are dependent on ASCL1 for proliferation^51–53^. Whether ASCL1 upregulation is required during the transition from PRAD to NEPC progression is unknown. The reliable kinetics of disease progression in the RPM model, coupled with the flexibility to perform multiplexed genome editing, allowed us to assess the requirement of *Ascl1* for NEPC transformation through CRISPR-mediated loss of function in RPM organoids (hereafter *Ascl1*^KO^; Supplementary Table 9). We compared the growth and histological features of *Ascl1*^wt^ versus *Ascl1*^KO^ RPM tumors following OT or subcutaneous (SQ) transplantation (Fig. 6a–d). *Ascl1*^wt^ RPM mice developed PRAD which progressed to NEPC over 6–10 weeks. This transition also occurred following SQ transplantation, indicating that the in vivo signal that triggers lineage plasticity is not restricted to the prostate microenvironment. The TME of these SQ tumors shared similar features seen in OT tumors by mIF (Supplementary Fig. 6a–c). In stark contrast, all *Ascl1*^KO^ RPM tumors (OT and SQ) developed PRAD with moderate to well-differentiated glandular histology, slower growth kinetics than *Ascl1*^wt^ RPM tumors and, most notably, no evidence of NE transformation (Fig. 6b–f and Extended Data Fig. 8a–f). Additionally, metastases were absent in *Ascl1*^KO^ RPM mice, compared to 50% incidence in *Ascl1*^wt^ RPM mice, despite comparable end-stage primary tumor weights at the OT site in intact and castrated hosts (Fig. 6g and Extended Data Fig. 8g). Thus, *Ascl1* is obligately required for the NEPC transition and metastasis in the RPM model.

We and others previously found that perturbations preventing lineage plasticity may restore sensitivity to androgen deprivation therapy in prostate cancer^16,53^. To address whether this is true in the context of *Ascl1* loss, we compared the tumorigenicity and histologic features of *Ascl1*^wt^ and *Ascl1*^KO^ RPM tumors following OT or SQ injection into intact versus castrated mice. *Ascl1*^KO^ tumors grew slower in castrated versus intact hosts in both the OT and SQ settings, despite the link between *RB1* and *TP53* loss and castration-resistance in multiple prostate models and in patients (Fig. 6b and Extended Data Fig. 8a–f). To distinguish between effects of castration on tumor engraftment versus tumor maintenance, we initiated chemical castration therapy (degarelix) in established SQ tumors (≥150 mm^3^). Notably, one castrated *Ascl1*^KO^ RPM mouse developed a tumor with chondrocyte-like histology, reminiscent of phenotypes in RPM-driven *Ascl1*^KO^ SCLC mouse models^52^ (Extended Data Fig. 8j). Degarelix treatment abrogated the growth of *Ascl1*^KO^ RPM tumors and extended survival, whereas *Ascl1*^wt^ RPM tumors were marginally impacted (Fig. 6c and Extended Data Fig. 8h,i). Degarelix treatment increased the fraction of NEPC tumor area and ASCL1^+^ tumor cells and decreased the fraction of AR^+^ tumor cells relative to vehicle treated RPM SQ tumors (Extended Data Fig. 8k).

**Fig. 7:**
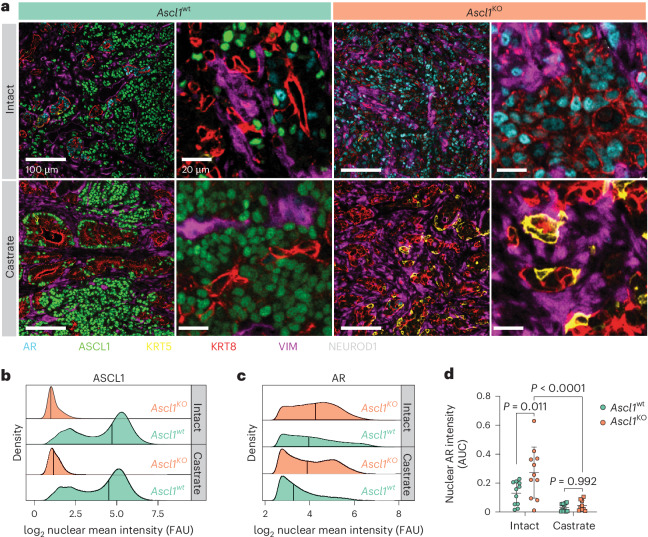
Loss of *Ascl1* results in enhanced AR expression and proportion of KRT8+ tumor cells. **a**, Representative confocal images of the tumors isolated from mice in Fig. 6d. Scale bars and pseudocolor legend indicated within the figure. Stains were repeated independently twice with similar results. **b**, Density plots of the log_2_(*x* + 1) transformed ASCL1 mean fluorescence intensity from all (OT and SQ) tumor cells. Tumor cells subset by all cells staining negatively for vimentin. Tumor genotype and treatment indicated in the figure panel. **c**, Density plots of the log_2_(*x* + 1) transformed AR mean fluorescence intensity from all OT tumor cells within the indicated genotypes and treatment groups. Tumor cells subset by all cells staining negatively for vimentin and positively for KRT8 and AR. **d**, Area under the curve (AUC) for all KRT8^+^:AR^+^ tumor cells (VIM^−^) across both SQ and OT tumors, containing a log_2_-transformed nuclear AR intensity score ≥3. Statistics derived using two-way ANOVA with Tukey’s multiple comparisons correction. Error bars indicate mean and s.d. Combined OT and SQ tumor sample sizes for all quantification and analysis performed in Fig. 7: *n* = 11 (*Ascl1*^wt^ and *Ascl1*^KO^ intact groups), *n* = 12 (*Ascl1*^wt^ castrate group), *n* = 9 (*Ascl1*^KO^ castrate group). FAU, fluorescence arbitrary unit. Source data

**Extended Data Fig. 9:**
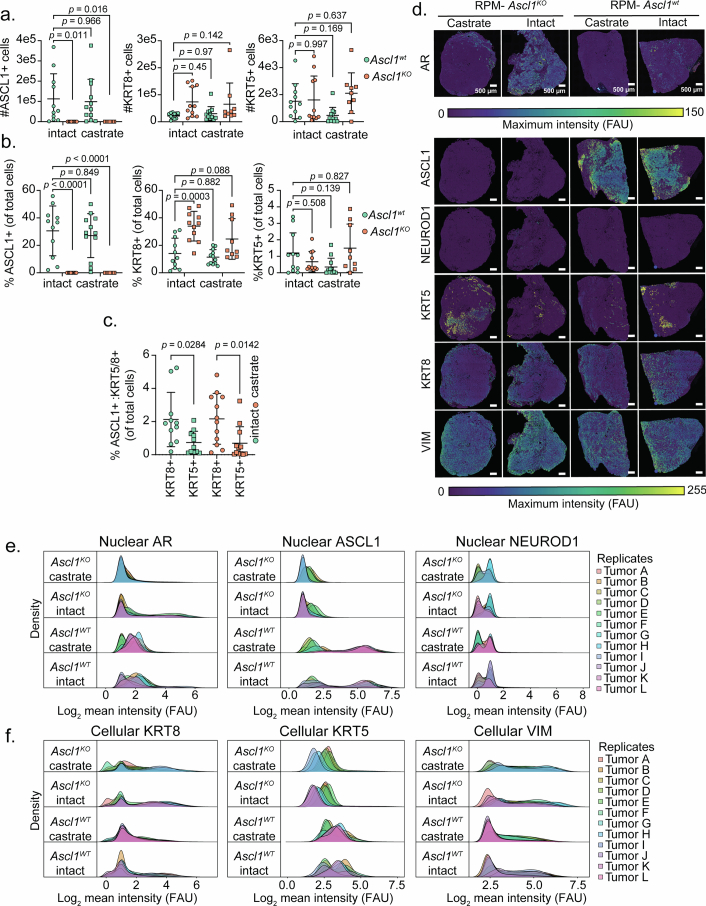
*Ascl1* loss prior to the NE transition promotes development of PRAD with increased KRT8 and AR expression. **a**. Number of ASCL1+ nuclei (left), KRT8+ cells (middle) and KRT5+ cells (right) in the indicated genotypes (legend on right hand-side) and treatment groups. **b**. Percentage of ASCL1+ nuclei (left), KRT8+ cells (middle) and KRT5+ cells (right) relative to total cells (DAPI+ nuclei) in the indicated genotypes (legend on right hand-side) and treatment groups. **c**. Percentage of ASCL1+ cells (DAPI+ nuclei) co-expressing either KRT8 or KRT5 within RPM-*Ascl1*^*WT*^ tumors in either intact or castrated hosts. Statistics in panels **a**-**c** derived from two-way ANOVA with Tukey’s multiple comparisons correction. Error bars in panels **a**-**c** denote mean and standard deviation. For **a**-**c**, data represents the average number of cells positive for the indicated marker across biologically independent tumors of the defined genotype and treatment cohort. *Ascl1*^*WT*^ intact, *n* = 11; *Ascl1*^*WT*^ castrate, *n* = 11; *Ascl1*^*KO*^ intact, *n* = 12; *Ascl1*^*KO*^ castrate, *n* = 9 tumors. **d**. Field of view images depicting maximum intensity score for all segmented cells within representative RPM tumors of the indicated genotype and treatment group. FAU = fluorescence arbitrary units. Data representative of *n* = 10 tumors. **e**. Density plots of the log_2_(x + 1) transformed mean fluorescence intensity for each nuclear protein. Each density plot represents signal intensity of tumor cells in tumor sections. **f**. Density plots of the log_2_(x + 1) transformed mean fluorescence intensity for each cytoplasmic protein. Each density plot represents signal intensity of tumor cells across *n* = 12 tumors. Source data

To investigate why tumors with *Rb1* and *Trp53* loss display increased androgen dependence in the context of *Ascl1* loss, we examined the expression of AR and luminal (KRT8) and basal (KRT5) cytokeratins. Consistent with their well-differentiated glandular morphology, RPM-*Ascl1*^KO^ tumors were dominated by AR^+^;KRT8^+^ tumor cells (Fig. 7a and Extended Data Fig. 9a–c). Furthermore, nuclear AR staining intensity was significantly elevated in *Ascl1*^KO^ relative to *Ascl1*^wt^ RPM tumors (Fig. 7b–d and Extended Data Fig. 9d–f). Taken together, these data suggest that *Ascl1*^KO^ RPM tumors are constrained to a luminal AR-dependent state.

### Ascl1 loss in established NEPC promotes tumor heterogeneity

**Fig. 8:**
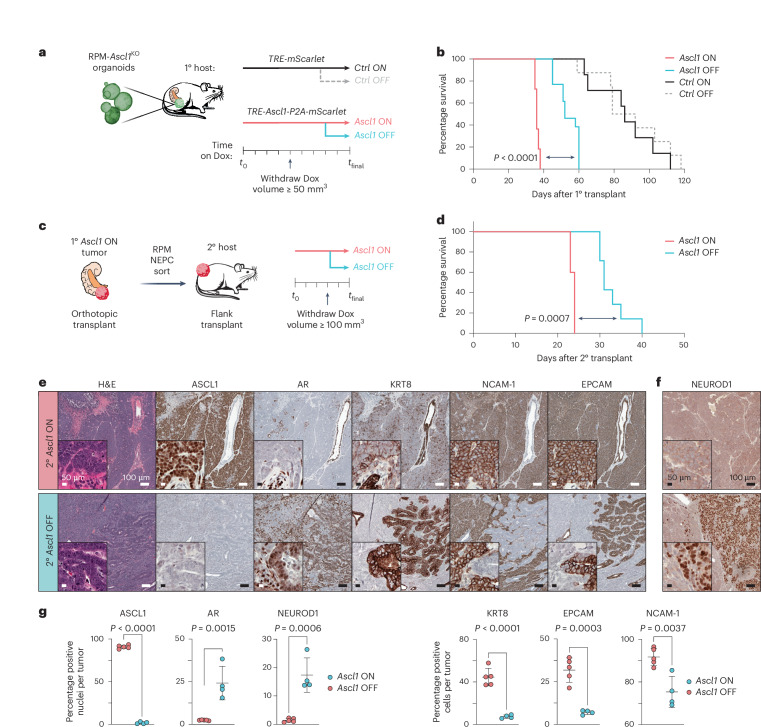
Loss of *Ascl1* in established NEPC results in modest tumor control and increased tumor heterogeneity. **a**, Schematic of *Ascl1* Dox-inducible in vivo platform. RPM-*Ascl1*^KO^ organoids infected with inducible *mScarlet* (*Ctrl*) or *Ascl1*-*P2A-mScarlet* (*Ascl1*) vectors were transplanted OT into mice fed Dox chow (primary recipient host, 1^o^). Tumor volume was monitored by ultrasound. Upon primary tumor establishment, mice were randomized into Dox ON (maintained) or Dox OFF (withdrawal) groups. **b**, Survival curves of *Ctrl* or *Ascl1* induced OT tumors following Dox maintenance (ON groups) or withdrawal groups (OFF groups). Statistics were derived from a log-rank (Mantel–Cox) test comparing primary *Ascl1* ON to primary *Ascl1* OFF groups. *Ctrl* ON *n* = 7, *Ctrl* OFF *n* = 8, *Ascl1* ON *n* = 11, *Ascl1* OFF *n* = 13 mice. **c**, Schematic of SQ *Ascl1* Dox-inducible in vivo platform (secondary recipient host, 2^o^). *Ascl1 ON* primary tumors were dissociated for flow cytometry to enrich for RPM-NEPC cells used for transplantation assays into the flanks of secondary recipient mice fed Dox chow. Tumor volume was monitored by caliper. Upon tumor establishment, mice were randomized into Dox ON (maintained) or Dox OFF (withdrawal) groups. **d**, Survival curves of *Ctrl* or *Ascl1* induced secondary tumors following Dox maintenance (ON groups) or withdrawal groups (OFF). Statistics were derived from a log-rank (Mantel–Cox) test. *Ascl1* ON *n* = 5 and *Ascl1* OFF *n* = 7 mice per group. **e**, Serial sections from secondary transplanted mice (SQ) stained for the indicated markers by H&E and IHC. **f**, Representative NEUROD1 IHC within a secondary transplant containing mostly NEPC histology. Data in **e** are representative of *n* = 5 tumors. Stains were repeated independently twice with similar results. **g**, Average percent marker positive nuclei (left) or cells (right) across 2^o^ SQ *Ascl1* ON (*n* = 5) or OFF (*n* = 4) tumors. Statistics are derived from a two-sided Student’s *t*-test. Error bars indicate mean and s.d. Scale bars are depicted in the figure panels. Source data

**Extended Data Fig. 10:**
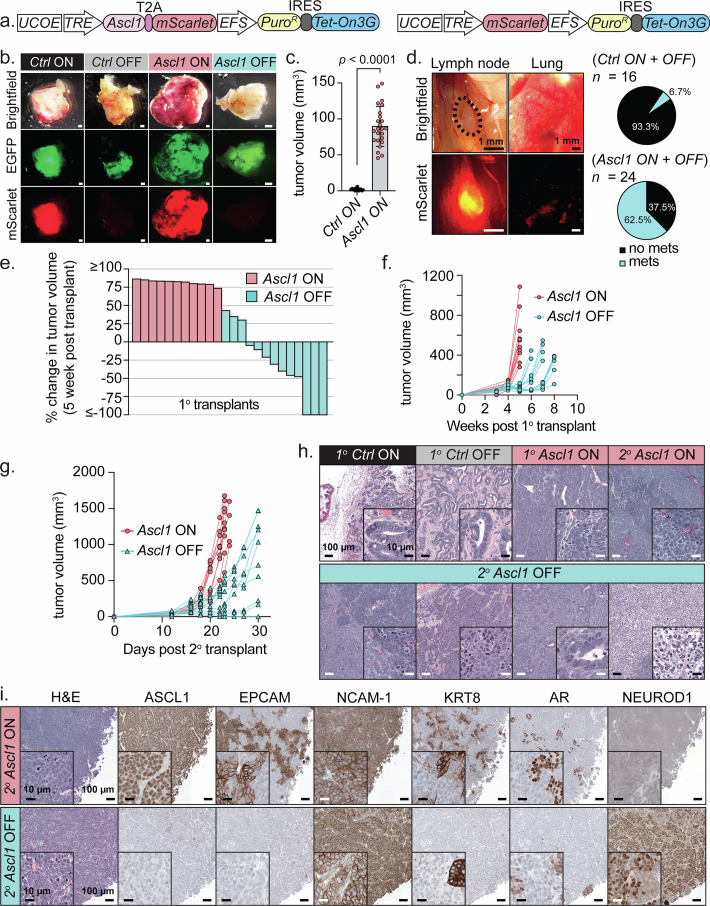
*Ascl1* loss in established NEPC promotes tumor heterogeneity. **a**. Schematic of the dox-inducible lentiviral vector used within this conditionally overexpress (left) *Ascl1-P2A-mScarlet* or (right) *mScarlet* in RPM-*Ascl1*^*KO*^ organoids. **b**. Representative stereoscopic images (brightfield and fluorescent) of OT tumors isolated from the indicated dox-maintained or withdrawn conditions. Scale bar represents 1 mm. Images are representative of *Ctrl* ON *n* = 7, *Ctrl* OFF *n* = 8, *Ascl1* ON *n* = 11, *Ascl1* OFF *n* = 13 tumors. **c**. Tumor volumes determined by ultrasound 4 weeks post OT of the indicated groups. All mice were maintained on dox chow. Statistics derived from two-sided Student's *t*-test and error bars denote mean and s.d. *Ctrl* ON *n* = 15; and *Ascl1* ON *n* = 24 tumors. **d**. (Left) Representative stereoscopic images (brightfield and fluorescence) of the draining lymph nodes and lungs of mice bearing OT *Ascl1* ON tumors. (Right) Pie charts indicating frequency of regional or distal micro-metastatic dissemination. Mouse sample size indicated in the figure legend. **e**. Percent change in primary recipient (1^o^) OT tumor volume measurements between 4–5 weeks of *Ascl1* ON, as determined by ultrasound. *Ascl1* OFF mice have been withdrawn from dox chow for 1 week. **f**. Longitudinal primary OT tumor volumes determined by ultrasound for the indicated groups. For panels **e**-**f**, *Ascl1* ON *n* = 11; and *Ascl1* OFF *n* = 13 mice. **g**. Longitudinal secondary recipient (2^o^) SQ tumor volumes determined by caliper for the indicated groups. *Ascl1* ON and OFF *n* = 10 tumors. **h**. Representative H&E images of the indicated groups spanning both primary recipient (OT) and secondary recipient (SQ) transplanted mice. **i**. Representative serially sectioned tumors stained for H&E and IHC of the indicated markers across *Ascl1* ON and *Ascl1* OFF secondary transplant recipient mice (SQ). Histology and IHC representative of tumors quantified in panels **a**-**g**. Stains were repeated *n* = 2 times with similar results. Images displayed represent regions maintaining NEPC histology and high fraction of NCAM-1 marker expression. All scale bars depicted in the figure panels. Source data

Given the crucial role of *Ascl1* in the NEPC transition, we next asked whether *Ascl1* is also required for the maintenance of established RPM-NEPC. To address this, we introduced a doxycycline (Dox) regulatable *Ascl1* cDNA (with a *cis*-linked *mScarlet* reporter allele) into RPM-*Ascl1*^KO^ organoids and performed OT in mice receiving Dox (Fig. 8a and Extended Data Fig. 10a). As expected, mScarlet^+^ OT primary tumors developed rapidly (within 5 weeks) in mice transplanted with RPM-*Ascl1*^KO^ organoids harboring the Dox-*Ascl1* allele (hereafter *Ascl1*^ON^), whereas tumors in mice transplanted with RPM-*Ascl1*^KO^ organoids containing the Dox-*mScarlet* allele (*Ctrl*^ON^) were delayed (Fig. 8b and Extended Data Fig. 10b,c). *Ascl1*^ON^ mice also developed metastases, whereas *Ctrl*^ON^ mice did not (Extended Data Fig. 10d), fully recapitulating the findings reported earlier (Fig. 6g).

After confirming the fidelity of the Dox-*Ascl1* rescue allele, we asked whether ASCL1 is required for sustained growth of *Ascl1*^ON^ tumors in a second cohort of *Ascl1*^ON^ and *Ctrl*^ON^ mice. These mice received Dox until tumors were established (≥100 mm^3^) followed by Dox withdrawal (hereafter *Ascl1*^OFF^ and *Ctrl*^OFF^; Fig. 8a and Extended Data Fig. 10b). Consistent with evidence that *ASCL1*-knockdown delays the growth of human NEPC xenografts^53^, most *Ascl1*^OFF^ tumors regressed within 1 week of Dox withdrawal but resumed growth within 2–3 weeks. Although short lived, Dox withdrawal provided a statistically significant survival benefit (log-rank Mantel–Cox test, *P* < 0.0001; Fig. 8b and Extended Data Fig. 10e, f).

To understand the mechanism of relapse after Dox withdrawal, we examined the histologic features and lineage of relapsed *Ascl1*^OFF^ tumors. To avoid PRAD contamination within RPM primary transplants (recall that RPM tumors retain mixed PRAD and NEPC histology), we focused solely on NEPC cells by isolating a pure population of RPM-NEPC from primary *Ascl1*^ON^ OT tumors, then retransplanting these cells SQ into secondary recipients (Fig. 8c, Supplementary Fig. 7a and Methods). As expected, the SQ transplants mirrored OT results: *Ascl1*^ON^ tumors grew rapidly, whereas *Ascl1*^OFF^ tumors grew slower with a significant increase in survival (log-rank Mantel–Cox test, *P* = 0.0007; Fig. 8d and Extended Data Fig. 10g). *Ascl1*^OFF^ tumors lacked nuclear ASCL1 expression, as expected (Fig. 8e). While some regions reacquired PRAD histological features (moderate-to-well-differentiated adenocarcinoma harboring KRT8 and AR expression), the predominant histology was high-grade ASCL1^−^ NEPC with sarcomatoid-like features (Fig. 8e–g, Extended Data Fig. 10h and Supplementary Fig. 7b–d). In contrast to NEUROD1^−^ RPM tumors discussed earlier (Fig. 2b), we now observed several NEUROD1^+^ NEPC regions (Fig. 8f, g and Extended Data Fig. 10i). In summary, while *Ascl1* is critical for initiating NE plasticity, established NEPC can circumvent *Ascl1* dependency, revealing unique pathologies and marker profiles not seen previously in RPM or RPM-*Ascl1*^KO^ tumors, indicative of selective pressure to maintain the NE state through upregulation of NEUROD1 and potentially other unidentified TF programs.

## Discussion

Lineage plasticity in cancer is a dynamic process that evolves over time. To gain a precise understanding of the underlying molecular events, a model amenable to repetitive interrogation and rapid perturbation is needed, ideally with reconstitution of the full repertoire of cells found within the TME. By integrating organoid techniques, genome engineering and in vivo transplantation, we generated a scalable, flexible and robust platform that mirrors the PRAD-to-NEPC transition with high fidelity. As with human NEPC, the mouse NEPC transition is accelerated by castration. At least two steps are required for plasticity to develop: *Rb1* loss, which we postulate primes cells for lineage transformation, followed by a second TME signal that ‘triggers’ upregulation of *Ascl1* and other lineage-defining TFs needed to complete the NEPC transition. Detailed characterization of the chromatin state of tumor cells before and during the lineage transition should shed light on the underlying molecular events driving NEPC fate^54^.

Application of spatial methods to this model provided insight into the origin of NEPC and its subsequent evolution. For example, the earliest detectable ASCL1^+^ cells often coexpress KRT8 or are adjacent to KRT8^+^ epithelial cells. In addition to implicating luminal cells as a likely cell of origin, this may provide a clue as to the source of the TME signal. Spatial analysis also allowed us to track expansion of ASCL1^+^ cells following the initial lineage transformation event, where NEPC regions gain expression of additional neural lineage development-associated TFs. NEPC evolution is associated with substantial changes in the TME, including near complete loss of mesenchymal cells, infiltrating CD8^+^ T cells and CD4^+^ T_reg_ cells.

The platform is amenable to multiplexed gene editing at the time of tumor initiation, allowing us to establish the critical role of ASCL1 in NE transformation in a matter of months (versus 1–2 years required for multigenic crosses using GEMMs). ASCL1 is a known dependency in SCLC, likely a consequence of tumor initiation in pre-existing ASCL1^+^ NE cells^51,52^. By contrast, ASCL1 is not expressed in prostate cancer until initiation of the NE fate transition. Previous work has shown delayed growth of ASCL1-expressing human xenograft models following *ASCL1* knockdown^53^. The dynamic nature of our platform documents an essential role of *Ascl1* in initiating the transformation of PRAD to NEPC. Through use of a Dox-inducible rescue alleles, the model can also address dependencies once NEPC is fully established. Unlike the transition phase, *Ascl1* extinction within established NEPC resulted in transient tumor regressions followed by ASCL1^−^ NEPC progression, underscoring the importance of early pharmacological intervention to prevent plasticity. Though direct inhibition of ASCL1 is challenging, therapies targeting the downstream target gene DLL3, including bispecific T cell engagers and other radioligand-based approaches, show clinical promise^55–57^.

The immunocompetent setting used in this model allows unresolved topics regarding the immunobiology of prostate cancer to be addressed. In contrast to cell lines derived from tumors that have escaped immune suppression, the immune-evasive mechanisms in the current model develop without any pre-transplantation immune-mediated selective pressure. This scenario allows deeper analysis of the earliest steps in immune escape and may shed light on novel immunity-bolstering strategies before tumors become depleted of T cells. Indeed, our spatial analysis shows that CD8^+^ T cells are present early in PRAD but absent in NEPC. The biology of these cells can be explored using model tumor antigens, combined with tetramer-based monitoring of T cell responses and selective depletion of specific myeloid and T_reg_ subpopulations.

Though our focus is on prostate cancer, the platform can be adapted to other epithelial lineages in which short-term organoid culture and OT methods have been developed. One disease that closely approximates the lineage transitions observed in prostate cancer is *EGFR*- or *ALK*-mutant lung adenocarcinoma, where NE transition is a mechanism of escape from EGFR or ALK inhibition^7,8,11,58,59^. *KRAS*^G12C^-mutant lung adenocarcinoma is a second example where transition to squamous histology can occur under the selective pressure of RAS inhibitor therapy^10,60^. Applications in bladder, pancreas, breast and gastrointestinal cancer can also be envisioned. In closing, we report a robust, scalable platform for studying lineage plasticity in a format amenable to deep molecular interrogation and perturbation and identify *Ascl1* as a critical gatekeeper of NE transformation and tumor heterogeneity in prostate cancer.

## Methods

### Ethics statement

All animal studies and procedures were approved by the Memorial Sloan Kettering Cancer Center (MSKCC) Institutional Animal Care and Use Committee (IACUC, protocol 06-07-012). MSKCC guidelines for the proper and humane use of animals in biomedical research were followed. The maximal tumor burden permitted by MSKCC IACUC of 2 cm^3^ was not exceeded in this study. Informed consent was obtained for all patient samples and approved by MSKCC’s Institutional Review Board (ref. 21-005; ClinicalTrials.gov NCT01775072).

### Animals

Male, 8–12-week-old C57BL/6J mice were maintained under pathogen-free conditions with a 12-h light–dark cycle, temperature controlled (20–25 °C) and 30–70% relative humidity. Food and water were provided ad libitum. Transplantation into immunocompetent hosts was performed on mice harboring conditional EGFP alleles to tolerize against EGFP-derived antigens (Jax, 026179). For organoids harboring Dox-inducible constructs, *Prkcd*^KO^ mice (Jax, 001913) were used to avoid rtTA-mediated rejection. All mice received pre- and postoperative analgesia (meloxicam and buprenorphine) and were monitored for signs of discomfort. At the end point, euthanasia was performed by CO_2_ asphyxiation followed by cervical dislocation.

#### Orthotopic prostatic organoid transplantation

For RPM and PtPM organoid transplants, 8–12-week-old EGFP-tolerized male mice were randomized for surgical implantation into a single dorsal prostatic lobe as described previously^21^. For *Ascl1*-inducible organoids, 1 × 10^5^ RPM-*Ascl1*^KO^ organoids harboring Dox-inducible *Ascl1-P2A-mScarlet* or *mScarlet* alone were injected into the prostates of *Prkcd*^KO^ mice. Dox chow (Inotiv, 0.625 g kg^−1^) was started 1 week before transplantation. Mice were randomized into *Ascl1*^ON^ (Dox maintained) or *Ascl1*^OFF^ (Dox withdrawn) cohorts when tumor volumes reached ≥50 mm^3^, as measured by small animal ultrasound (Fujifilm-Visualsonics, Vevo2100). Tumor volumes were calculated using Vevo Lab Software (v.5.9.0).

#### Subcutaneous prostatic organoid transplantation

For allograft experiments, 2.5 × 10^5^ single-cell suspension of organoids in 100 μl 50% Matrigel were injected into the depilated flanks of EGFP-tolerized mice. For secondary transplants, 5-week *Ascl1*^ON^ OT tumors were processed to a single-cell suspension (FACS buffer: 0.5% BSA and 1 mM EDTA) and sorted (Sony MA900, 100-μm sorting chip, Sony Biotechnology, LE-C3210) for 4,6-diamidino-2-phenylindole (DAPI)^−^, EGFP^+^, mScarlet^+^, EPCAM^+^ and NCAM-1^+^ cells (for gating strategy, see Supplementary Fig. 7a). Antibodies used for flow cytometry listed in Supplementary Table 10. After sorting, 1 × 10^5^ cells were injected into the flanks of secondary Dox chow pre-fed *Prkcd*^KO^ mice as described above. Mice were randomized into *Ascl1*^ON^ or *Ascl1*^OFF^ groups when tumors reached ≥150 mm^3^. At the experimental end point, secondary tumors were collected for formalin-fixing paraffin-embedding (FFPE) or processed for flow cytometry (Supplementary Fig. 7b–d). Subcutaneous tumor volumes were measured by caliper and calculated as previously described^61^.

#### Castration studies

Mice with orthotopic prostate tumors were randomized to castration or sham surgeries 2 weeks after surgery. Then 1 × 10^6^ RPM-*Ascl1*^wt^ or RPM-*Ascl1*^KO^ organoids were injected into the flanks of immunocompetent mice. Mice were randomized into vehicle (5% d-mannitol, Sigma M4125) or Degarelix (15 mg kg^−1^, Sigma SML2856) groups once tumors measured ≥150 mm^3^, with treatments given subcutaneously every 14 days (100 μl). Tumor volumes were measured as described above. At time of euthanasia, serum testosterone levels were assessed by ELISA (Abcam, ab285350).

### Human specimens

Informed consent was obtained for all patients and approved by MSKCC’s Institutional Review Board 21-005 (NCT01775072). A prostate tumor specimen was collected from a 62-year-old male with localized PRAD undergoing XRT followed by salvage prostatectomy after ADT and docetaxel. The tumor in the bladder arose by extension of a prostate tumor recurrence in the surgical bed. Pathological review revealed small cell carcinoma arising from PRAD, with tumor cells showing focal positivity for SYP, CHGA (patchy) and weak focal PSA and PSMA. Tumor sample was sectioned and processed for COMET-based mIF using the antibodies listed in Supplementary Table 10.

### Lentiviral production

Lentiviruses were produced by co-transfection of 293T cells (Takara, 632180) with lentiviral backbone constructs and packaging vectors (psPAX2 and pMD2.G; Addgene, #12260 and #12259) using TransIT-LT1 (Mirus Bio, MR 2306) and concentrated by ultracentrifugation as previously described^61^.

### Molecular cloning

Lentiviral vector (LVt-UBC-cMYC-P2A-EGFP) was generated using Gibson assembly. PCR fragments were amplified containing UBC promoter, *cMyc*^T58A^ codon optimized cDNA (geneblock, IDT) and a *P2A-EGFP* sequence, mixed within a Gibson master mix reaction and transformed into Stbl3 chemically competent *Escherichia* *coli* (Thermo, C737303). All plasmids were purified (QIAGEN, 12943) and sequence-validated through long-read sequencing (SNPsaurus). Lentiviral construct UT4GEPIR (Addgene, #186712) was used for cloning Dox-inducible *Ascl1* and *mScarlet* constructs. *Ascl1-T2A-mScarlet* and *mScarlet* geneblocks were cloned with BamHI and SceI overhangs.

### Organoid culture

Organoids were derived, cultured, and infected as previously described^19,20^. Ribonucleoprotein (RNP) electroporation was performed as previously described^21^. Sequences for sgRNAs used can be found in Supplementary Table 11. For monolayer adaptation, 2 × 10^6^ single-cell organoid suspensions were seeded into 10-cm collagen-coated plates (Corning, 356450) and expanded in standard mouse prostate organoid medium supplemented with 10 μM Y-27632 (Tocris, 1254) for 5 days and then collected for transplantation or western blot validation. Of note, FBS was avoided throughout all steps of organoid culture, as FBS promotes differentiation and cell death.

### RNA isolation from organoid cultures and bulk tumors

Tumors were isolated and validated for EGFP fluorescence (Nikon, SMZ18). and lysed in 250–500 μl RLT buffer supplemented with β-mercaptoethanol using ceramic beads (MP, 6910500) and loaded onto a Fisher Bead Mill 24 (1-min intervals on ice until fully lysed). RNA was isolated using QIAGEN RNeasy kit (QIAGEN, 74106). Organoids were similarly dissociated, resuspended in RLT buffer with β-mercaptoethanol, and disrupted with a Qiashredder (QIAGEN, 79656) before RNA isolation using the same kit. For qPCR, purified RNA was reverse transcribed (Thermo, 4368814) and quantified (Applied Biosystems, QuantStudio 6) with SYBR green reagent (Thermo, A46110). See Supplementary Table 11 for primer sequences used for qPCR.

### Immunohistochemistry

Samples were fixed followed by hematoxylin and eosin (H&E) or chromogenic immunohistochemistry (IHC) staining as previously described^61^. Antibodies used for IHC are listed in Supplementary Table 10. Slides were scanned on a Pannoramic Scanner (3DHistech) with a ×20/0.8 NA objective and visualized in ImageJ or QuPath (v.0.4.2).

### Multiplexed immunofluorescence

Samples were pretreated with EDTA-based epitope retrieval (ER2, Leica, AR9640) for 20 min at 95 °C. Staining and detection were conducted sequentially using antibodies listed in Supplementary Table 10. After 1 h incubation, Leica Bond Polymer anti-rabbit HRP was applied, followed by Alexa Fluor tyramide conjugate 488 and 647 (Life Technologies, B40953, B40958) or CF dye tyramide conjugate 430, 543, 594, and 750 (Biotium, 96053, 92172, 92174 and 96052) for signal amplification. Epitope retrieval was repeated between rounds to denature antibodies before applying the next primary antibody. Slides were counterstained with DAPI (5 μg ml^−1^, Sigma Aldrich), rinsed in PBS and mounted in Mowiol 4–88 (Calbiochem). Slides were scanned on a Pannoramic Scanner (3DHistech) with a ×20/0.8 NA objective and visualized in ImageJ or QuPath. Confocal microscopy was performed on a Leica Stellaris 8. For sequential immunofluorescence (Lunaphore COMET), the tissue was cut to 4 μm onto positively charged glass slides. Slides were baked at 64 °C for 1 h. Dewaxing and antigen retrieval was performed on the Leica Bond RX with 30-min retrieval in ER2 solution. Slides were washed 3× for 1 min in DI water and loaded onto instrument. The 20-plex antibody panel can be found in Supplementary Table 5.

### Immunoblotting

Single-cell organoid suspensions or monolayer cells were lysed in 125–250 μl ice-cold RIPA (Pierce, 89900) supplemented with 1× Complete Mini inhibitor mixture (Roche, 11836153001) and processed further for SDS–PAGE as previously described^61^. Antibodies used for western blots are listed in Supplementary Table 10. Blots were developed in Amersham ECL western detection region (Cytiva, RPN2236) and imaged on a Cytiva Amersham ImageQuant 800.

### Isolation and validation of *Ascl1* knockout organoid clones

*Ascl1* sgRNA-targeted RPM organoids with Cas9 RNP were expanded for 5 days, then gently triturated in 0.5% BSA in PBS. Intact spheres were isolated using a 20 μl pipet, expanded, and genomic DNA was extracted (QIAGEN, 69506) for *Ascl1* target locus amplification with the KAPA mouse genotyping kit (Fisher Scientific, 50-196-5243; see Supplementary Table 11 for PCR primers sequences). The 170-bp PCR product was purified (QIAGEN, 28706) and submitted for library preparation and sequencing at the Integrated Genomics Operation at MSKCC. Barcoded libraries were pooled and sequenced on NovaSeq 6000 (PE150, Illumina NovaSeq 6000 S4 Reagent kit, 300 cycles) with an average number of 1.3 M reads per sample. Alignment and modification quantification were performed with CRISPResso2 (http://crispresso.pinellolab.org/) using default parameters. Six sequence-validated biallelic RPM-*Ascl1*^KO^ clones were pooled and expanded for transplantation experiments.

### snRNA sequencing and sample preparation

Dissected tumor samples were sliced into ~5 × 5-mm pieces and flash frozen. Nuclei were isolated using the Singulator 100 (S2 Genomics) and standard nuclei-isolation protocol supplemented with 3.5 μl 1 M dithiothreitol (Sigma, 43816) and 87.5 units of Protector RNase inhibitor (Sigma, 3335402001). The suspension was cleaned with sucrose density gradient (Sigma, NUC201-1KT) and centrifuged at 500*g* for 5 min. Nuclei were resuspended in wash buffer (10 mM Tris-HCl, pH 7.4, 10 mM NaCl, 3 mM MgCl_2_, 1 mM dithiothreitol, 1% BSA and 1 U μl^−1^ Protector RNase inhibitor), filtered through a 35-μm strainer and sorted (7-AAD^+^) to obtain a single-nuclei suspension. Nuclei were processed (Single Cell Analytics Innovation Lab, MSKCC) on a Chromium instrument (10x Genomics) for 3′ v.3.1 snRNA-seq, following manufacturer protocols. After capture, complementary DNA was purified with Dynabeads (Thermo, 37012D) and amplified per manual instruction. Libraries targeting ~10,000 cells per sample were sequenced on an Illumina NovaSeq S4 (R1, 28 cycles; i7, 8 cycles; R2, 90 cycles; Integrated Genomics Operation, MSKCC). FASTQ files were processed using 10x Cell Ranger v.6.1.2 to align reads to the mm10/GRCm38 reference genome, including *Myc-P2A-EGFP* transgene sequences (Extended Data Fig. 1b). Introns were included to account for the higher rate of intronic reads in snRNA and feature-barcode matrices were generated for subsequent analysis.

### Bulk RNA-seq and analysis

RNA was quantified and quality controlled via Agilent BioAnalyzer and 500 ng total RNA followed by poly-A selection library preparation (RIN 8.3–10, TruSeq Stranded mRNA LT kit, RS-122-2102). Sequencing was performed on a NovaSeq 6000 (PE100, Illumina SX Reagent kit), yielding an average of 24 million paired reads per sample. Bulk RNA-seq analysis was performed using the Seq-N-Slide pipeline (https://github.com/igordot/sns), through *rna-star* followed by rna-star-groups-dge routes with quality control assessment (MultiQC^62^, Python/cpu v.2.7.15) and adaptor trimming (Trimmomatic^63^, v.0.36). Reads were aligned to the mm10/GRCm38 mouse reference genome with a splice-aware^64^ (STAR v.2.7.3a) alignment, followed by featureCounts^65^ (subread v.1.6.3) to generate an RNA counts table. Counts were normalized and tested for differential mRNA expression using negative binomial generalized linear models as implemented in DESeq2 (ref. ^66^) (v.1.40.1). The log_2_ fold changes of contrasts (*c*^*t*^*β*/√*c*^*t*^*Σc*) were shrunken following ‘lfcShrink(type = ‘ashr’)’ to stabilize genes with low or variable counts. Differential expression was assessed by principal-component analysis or unsupervised hierarchical clustering and visualized by a volcano plot and TPM expression heatmap. Differentially expressed genes (DEGs) were analyzed for gene set enrichment analysis (GSEA) with R packages: fgsea v.1.26.0 and msigdbr v.7.5.1 via the pre-ranked method with 10,000 permutations, based on the adaptive multilevel splitting Monte Carlo approach. DEGs were further analyzed for GSEA using curated NEPC signatures^16^ with variance-stabilized log_2_ fold changes calculated by DESeq2 as ranking metric. All *P* values were adjusted with the Benjamini–Hochberg method.

### Reclustering of the public scRNA-seq dataset

To derive gene sets for benchmarking BayesPrism, we used an independent scRNA-seq dataset from prostates derived from *Pten*^fl/fl^; *Rb1*^fl/fl^; *Trp53*^fl/fl^; *Probasin-Cre* (PtRP) GEMMs^16^. This dataset was generated using a mouse model that similarly transitions from PRAD to NEPC covering a broad diversity of cell types. We reclustered the data to improve the granularity of mesenchymal cells. To improve mesenchymal cell granularity, we reclustered GFP^−^ mesenchymal cells. We selected the top 3,000 highly variable genes using Scanpy’s pp.highly_variable_genes with raw counts, flavor seurat_v.3 and span = 1. Raw counts were normalized by the library size of each cell/10^4^, followed by log_2_(*X* + 1) transformation. We selected the top 30 principal components using scanpy.tl.pca, followed by clustering with Phenograph (*k* = 30, clustering_algo = ‘leiden’), generating 20 clusters. Clusters were annotated by markers from Niec et al.^33^, with endothelial, lymphatic, glial cells and pericytes/myofibroblasts clearly distinguishable (Supplementary Fig. 5a). The remaining clusters were annotated as Mes-1 and Mes-2 (Supplementary Fig. 5b,c).

### snRNA-seq analysis

For mouse snRNA-seq datasets, ambient RNA molecules were removed using CellBender^67^ with –expected-cells 5000 and –total-droplets-included 20,000. All downstream processing of snRNA-seq data and scRNA-seq data was performed in Scanpy^68^. Low-quality cells, unexpressed genes and potential doublets were excluded. Genes detected in fewer than three cells, cells with fewer than 200 genes or 1,000 unique molecular identifiers (UMIs) and cells with a mitochondrial fraction >10% were removed. Mitochondrial and ribosomal protein-coding genes were also omitted from downstream analysis. Doublets were identified using Scrublet^69^ retaining 4,872 cells with a median of 7,055.5 UMIs per cell. We selected the top 3,000 highly variable genes using Scanpy’s pp.highly_variable_genes with raw counts, flavor = ‘seurat_v3’ and span = 1. Raw counts were normalized by the library size of each cell/10^4^, followed by log_2_(*X* + 1) transformation. We selected the top 30 principal components using scanpy.tl.pca, followed by clustering with Phenograph^70^ (*k* = 30, clustering_algo = ‘leiden’), generating 19 clusters. Marker genes (Supplementary Figs. 2a and 5a–c) were used for cell typing. Malignant and nonmalignant cells were distinguished based on copy-number analysis inferred using inferCNV^71^ with myeloid and endothelial cells as the normal cell reference. Clusters were annotated based on copy-number variant alterations and marker gene expression, identifying them as normal epithelial and mesenchymal cells, as well as tumor subtypes, including NE, EMT and tumor luminal/basal (Supplementary Figs. 2a,b and 5a–c).

### Visium preparation

We generated Visium data from two adjacent sections as technical replicates from 10-week RPM tumors (*n* = 2 mice). Spatial gene expression slides were prepared using FFPE sections (Molecular Cytology Core, MSKCC) following the manufacturer’s recommended protocol (10x Genomics, 1000337). After real-time PCR evaluation, sequencing libraries were prepared with 11–14 PCR cycles, pooled equimolar and sequenced on a NovaSeq 6000 in (PE28/88 run, NovaSeq 6000 SP Reagent kit, 100-cycles, Illumina). FASTQ files were processed using spaceranger count (v.2.0.0) to align reads to the GRCm38 (mm10) reference genome and generate count matrices. Each sample yielded an average of 74 million paired reads, corresponding to 37,000 reads per spot.

### Analysis of Visium data

Visium data from two replicates were deconvolved using BayesPrism with the snRNA-seq data from the 10-week RPM tumor as the reference, following setups similar to those previously reported^33,72–74^.

To enhance the signal-to-noise ratio, we selected marker genes that are differentially upregulated in each of the 19 cell types, as defined by the 19 Phenograph clusters in the snRNA-seq data. We took the union of these marker genes and deconvolved over these genes. We performed a pairwise *t*-test using the ‘findMarker’ function from the scran package^75^, accessible via the wrapper function ‘get.exp.stat’ from BayesPrism. For each nontumor cell types (four total: mesenchymal, myeloid, normal luminal/basal and endothelial cells), we required the maximum *P* value to be <0.01 and the minimum log_2_ fold change to be >0.1 across comparisons with other cell type. For tumor cell type (15 in total: NE, EMT and tumor luminal/basal), the same thresholds were applied, but comparisons were only made against nontumor cell types to retain the maximum number of tumor-specific genes for PrismSpot analysis. This was achieved by defining a coarse-level cell-type labeling, where 15 tumor cell types were grouped into a single tumor cell type and using it as the ‘cell.type.labels’ argument, while setting the original 19 cell types ‘as cell.state.labels’ in BayesPrism’s built-in function ‘get.exp.stat’.

To speed up deconvolution we excluded genes expressed in fewer than four spots, resulting in 5,125 genes. Each of the 19 cell states was treated as an individual cell type when constructing the reference. The pseudo.min parameter was set to 0 to maximize the contrast between cell types. Other BayesPrism parameters were left as default, including a flat Dirichlet prior (*α* = 1), a Markov chain Monte Carlo chain length of 1,000 (with a 500-step burn-in) and a thinning set to 2. Updated Gibbs sampling was used for a robust batch effect correction between the snRNA-seq and the FFPE Visium data.

We benchmarked BayesPrism’s accuracy and robustness in deconvolving cell type fractions from Visium data. To estimate ground truth, we used marker genes and histology with H&E. BayesPrism’s inferred NEPC fractions showed strong concordance with histological regions (Fig. 5c). We assessed robustness by comparing cell type fractions across histologically defined regions between two technical replicates, adjusting regions manually to accommodate shifts on the *x*–*y* plane between technical replicates. For each region, we compute the average fractions for each cell type. We then computed both cell type-level and region-level Pearson’s correlation coefficients (Extended Data Fig. 7a,b).

### PrismSpot analysis

For the Hotspot analysis, we used spot-specific tumor gene expression by summing the posterior mean of cell type-specific gene expression (*z*) across all tumor clusters (*n* = 15) output by BayesPrism. We rounded up the posterior mean of *z* because Hotspot models raw count data using a negative binomial distribution. To define the neighborhood structure for spatial coexpression, we performed a single Hotspot analysis for all Visium spots while restricting neighborhoods within each mouse and technical replicate. We adjusted Visium spot coordinates for each tumor sample to prevent overlap among spots from different samples. Genes with zero count in all spots and spots with fewer than 1,000 genes or 1,000 UMIs were excluded. We set ‘n_neighbors = 6’ in Hotspot’s ‘create_knn_graph’ and treated all adjacent spots equally. We selected genes with a false discovery rate (FDR) of < 0.01 for spatial autocorrelation *z*-scores (Fig. 5). The TFs were clustered into modules using the ‘create_modules’ function, with parameters ‘min_gene_threshold = 15’, ‘core_only = True’ and ‘fdr_threshold = 0.05’, which performs a bottom-up clustering procedure by iteratively merging two genes/modules with the highest pairwise *z*-score.

To ensure robustness, we implemented a subsampling strategy, drawing 60% of the reads for each mouse tissue on each Visium slide 100 times. We reclustered genes into modules and a consensus score for each gene, representing the frequency of co-occurrence in the same module across subsampled datasets. Genes with an average consensus score ≥0.8 were selected as representative genes, resulting in 71 out of 181 genes from three out of the original five spatial modules (Fig. 5e and Supplementary Table 7).

### Benchmarking PrismSpot

We compared Hotspot results between PrismSpot and ‘standard’ Hotspot (using un-deconvolved raw counts), hereafter referred to as Hotspot, by analyzing autocorrelation and pairwise local correlation coefficients for marker genes from different cell types. Marker genes were derived from the GEMM scRNA-seq dataset^16^ mentioned above, grouped to match the granularity of the snRNA-seq reference. Specifically, we grouped Mes-1 and Mes-2 as mesenchymal; *Tff3*, mutant L1, mutant L2, mutant B1, NEPC-*Pou2f3* and NEPC as tumor; macrophages, neutrophils and DC as myeloid. We performed differential expression using a pairwise Student’s *t*-test between tumor, stromal, myeloid and endothelial cells, selecting markers with a maximum *P* value < 0.01 and minimum log_2_ fold change >0.1.

We performed one-sided paired Student’s *t*-tests to compare autocorrelation *z*-scores between PrismSpot and Hotspot, with null hypothesis tailored for tumor and nontumor cell types. For nontumor cell types (for example endothelial, myeloid and mesenchymal) we define null hypothesis H_0_: PrismSpot *z* > Hotspot *z*, while for tumor cells we define H_0_: PrismSpot *z* < Hotspot *z*. To ensure statistical power was similar in the comparison of autocorrelation scores for each cell type, we selected the top 100 genes that pass the threshold mentioned above based on log_2_ fold change. The *P* values were 4.0 × 10^−3^, 2.1 × 10^−6^, 6.0 × 10^−9^ and 3.8 × 10^−3^ for endothelial, myeloid, mesenchymal and tumor cells, respectively (Supplementary Fig. 4c).

Likewise, we performed one-sided paired Student’s *t*-tests to compare local pairwise correlation *z*-scores between PrismSpot and Hotspot across three categories: (1) between a pair of tumor marker genes; (2) between a tumor marker gene and a marker gene for nontumor cell types; and (3) between a pair of marker gene for nontumor cell types. As pairwise local correlation can be both positive and negative, we performed statistical tests on the absolute value of *z*-scores. For tumor versus nontumor and nontumor versus nontumor categories, we define null hypothesis H_0_: |PrismSpot| > |Hotspot|, whereas for the tumor versus tumor category, we define H_0_: |PrismSpot| < |Hotspot|. The *P* value of the tumor versus tumor category was 3.3 × 10^–5^. For the tumor versus nontumor and nontumor versus nontumor categories, *P* values were less than the numeric limit 2.2 × 10^−16^ (Supplementary Fig. 4g).

### Pathology annotation and spatial immunofluorescence analysis

Sections processed for H&E and mIF were reviewed by a board-certified genitourinary pathologist (A.G.). Graded histological areas were used to identify regions of PRAD and NEPC on serially sectioned samples processed for 10x Visium and mIF.

#### Cell segmentation

We utilized Mesmer^76^ (standard model), to identify cell boundaries in COMET images. The input to Mesmer is a single nuclear image (DAPI) and single membrane and/or cytoplasm image. To demarcate cell types, we merged images from multiple cell-type-specific membrane and/or cytoplasmic markers by applying min–max normalization (‘MinMaxScaler’, ‘sklearn.preprocessing’ package with default parameters) to each channel before summation. We combined CD45, CD20, CD4, CD8, CD11b, CD11c, Ly6G (immune cells), CD31 (endothelial cell), GFP, KRT8 (tumor epithelial cells), VIM and α-SMA (stromal cells). We ran Mesmer on these images with standard parameters to predict cell boundaries (modified to exclude cells ≤36 pixels) and calculated the cell size, eccentricity and centroid of each cell boundary. Images were preprocessed to half their size (to 0.46 µm per pixel) to accommodate system memory constraints (128 GB).

#### Normalization

We quantified raw per-cell marker expressions by aggregating pixel brightness within each cell boundary. To neutralize cell size variance, expressions per channel were normalized against cell boundary area. We found bimodal distributions of cell size and DAPI expression in our dataset. The lower mode of DAPI contained primarily empty regions and the upper mode of cell size contained cell segmentation errors. We then filtered all cells with DAPI values less than 2,096 (estimated from distribution) and cell size larger than 2,500 (estimated from distribution). The marker intensity signals then underwent logarithmic transformation.

#### Cell type identification

We used a clustering-based approach for tumor cell identification within our dataset. We constructed a 20-dimensional count matrix based on the computed per-cell normalized marker expression and then constructed a *k*-nearest graph (*k* = 30) based on similarity of marker expression using the Leiden algorithm^77^, yielding 27 distinct clusters. These clusters were labeled as stromal, tumor (marked by GFP, ASCL1 and KRT8) or artifacts (characterized by low marker expression), with artifact cells excluded from further analysis. Cell types were classified based on marker intensity distributions: lymphatic endothelial cells (LYVE1 > 7.5), blood vessel endothelial (CD31 > 8) and immune (CD45 > 8). The distribution patterns of this coarse classification are reported in Fig. 4a. With the same approach, we identified subpopulations in immune cells: CD4^+^ T cell (CD4 > 7), B cell (CD20 > 8), CD8^+^ T cell (CD8 > 8.3), T_reg_ (FOXP3 > 8), CD11b F4/80^+^ myeloid (Mac3; CD11b > 7 and F4/80 > 7), CD11b F4/80^−^ myeloid (Mac1; CD11b > 7 and F4/80 < 7), CD11c F4/80^+^ myeloid (Mac2; CD11c > 8.5 and F4/80 > 7).

#### Spatial analysis

To examine cellular organization within lymph node tissue, we constructed a spatial neighborhood graph by linking cells within a 20-pixel radius (~2.5 times the median cell radius). This graph contained an average of 4.5 neighbors per cell. A neighborhood enrichment matrix was generated to map enriched proximity between cell types, with axis labeling indicating source and neighboring cell types and matrix values representing total neighboring counts. Normalizing these counts by the total number of neighbors per cell type produced a cell proximity frequency matrix (Fig. 4g), showcasing the likelihood of each cell type neighboring another. Both were implemented using Squidpy^78^.

For infiltration analyses, we used HALO and HALO A.I. v.3.6.4134 (Indica Labs) for nuclear and cytoplasmic segmentation, trained on a set of control tumor slides. Segmentation was performed using the DAPI channel, with manual review and vetting to ensure high-quality segmentation across slides. Due to high cellular density in some tissues, we employed Mesmer-based segmentation for more robust results. Marker thresholding was kept consistent across slides, and DAPI-based criteria filtered single cells, including (1) nuclear DAPI measurements above user-input threshold; and (2) the nuclear/cytoplasmic ratio DAPI measurement was above a user-input threshold. Cells were assigned to cell types based on marker coexpression in a layered fashion (Supplementary Table 6). Infiltration metrics were calculated by binning tumor regions and normalizing cell counts per bin area. Tumors within an individual metastatic tissue sample were pooled for infiltration analysis. Cells within each bin were quantified and normalized to the bin area, and the mean cell density per bin was averaged across mouse samples. Replicate samples (*n* = 3 tumors) were used to calculate the standard error. Data were visualized in R using ggplot2 with Loess function smoothing. For histological characterization, a Random Forest tissue classifier was refined (HALO) using *n* = 10 primary and metastatic RPM tumors containing examples of NEPC, PRAD and stroma. An RPM (*n* = 1) tumor derived from a castrated host was used for chondrosarcoma histology training. Tissue classification was validated by pathologist, A.G.

### Analysis of human scRNA-seq PRAD and NEPC myeloid subsets

FASTQ files from a previously published single-cell dataset^16^ (histologically verified CRPC-PRAD (*n* = 9) and NEPC (*n* = 3)) was mapped to human reference genome GRCh38 using Cell Ranger v.7.0.1 to generate count matrices (transcripts and/or genes × cells). Downstream analyses were performed using the Seurat R package (v.4.4.0). Cells were removed if genes were not detected in at least ten cells. Cells were filtered based on the following: (1) fewer than 500 genes; and (2) ≥30% mitochondrial counts and ≤500 UMI counts. Putative doublets were removed using scDblFinder^79^. Combining samples from all CRPC-PRAD and NEPC sampled yielded 63,834 cells × 30,519 genes. To normalize the data, the ‘LogNormalize’ method was used with a pseudocount = 1 and scale factor = 10,000. The top 2,000 highly variable genes were identified using the ‘FindVariableFeatures’ function. fastMNN was utilized across all cell types to perform batch correction (using all 30,519 genes). A *k*-nearest neighbor graph was constructed using the ‘FindNeighbors’ function with the first 30 ‘MNN’ dimensions on the batch-corrected count matrix. Clustering was performed using the ‘FindClusters’ function based on the Louvain algorithm with a resolution of 0.3. The resolution value was determined to be 0.3 as it best matched the expression patterns of all lineage markers. The clustered cells were visualized using ‘RunUMAP’ with the first 30 dimensions from the dimensional reduction ‘MNN’, and the clusters expressing myeloid lineage markers (*CD14*, *LYZ* and *IL1B*) were identified with these cells being subsetted for downstream analysis (*n* = 7,004 myeloid cells). Reclustering with a resolution of 1 was then conducted these putative myeloid cells using the batch-corrected matrix (from the upstream correction) yielded 17 clusters. DEGs for each cluster were identified using ‘FindMarkers’ with the MAST algorithm (v.1.24.1) and thresholds of Bonferroni-adjusted *P* value <0.05 and log_2_fold change > 0.5. Of note, four clusters (*n* = 1,282 cells) had low UMI counts, one cluster (*n* = 382 cells) showed top DEGs possibly indicative of doublet cell types expressing markers for both epithelial cells and myeloid cells (*KLK3* and *CD14*) and one cluster (*n* = 42 cells) expressed high levels of proliferation-related genes (*MKI67*, *TOP2A* and *STMN1*) and therefore, were removed. This yielded a total of 5,298 myeloid cells (4,348 CRPC-PRAD and 950 NEPC cells). To identify the subtypes of TAMs, module scores from predefined gene sets for each TAM^80^ were used and scores were calculated using ‘AddModuleScore’. Cells were labeled based on the median and maximum signature scores per cluster.

### Analysis of human prostate SU2C dataset

The FPKM-normalized RNA-seq from Abida et al. was downloaded from https://github.com/cBioPortal/datahub/tree/master/public/prad_su2c_2019 (ref. ^48^). We selected patient samples sequenced by the poly-A enrichment protocol, as it contains more samples with histologically verified NEPC. *n* = 9 NEPC samples and *n* = 50 PRAD samples. We performed differential expression analysis to derive the log_2_ fold change and *P* values between NEPC and PRAD samples for each gene independently. We computed log_2_ ((mean expression of NEPC samples + 1)/(mean expression of PRAD samples + 1)). The statistical significance was computed using a two-sided Wilcoxon test.

### Statistics and reproducibility

Statistical analyses were performed using GraphPad Prism (v.9.5.1) or R (v.4.3.1). A *P* value < 0.05 was considered significant. Exact *P* values are reported in each figure unless *P* < 0.0001. Variance between compared groups was similar. A two-tailed Student’s *t*-test was used for comparisons between untreated to treated samples or between genotypes or tumor cell types. A one-way analysis of variance (ANOVA) with Sidak’s or Tukey’s correction was used for comparisons across multiple groups. For time-course experiments, a two-way ANOVA with multiple comparisons correction was applied. Figure legends denominate statistical analysis used. Sample sizes were not statistically predetermined but were similar to those in previous studies with the same type of experiments and readout^16,26,33^. Blinding was applied during group allocation, data collection and analysis. Animals without detectable tumors or with severely ulcerated tumors were excluded from the analysis; no other exclusions were made.

### Reporting summary

Further information on research design is available in the Nature Portfolio Reporting Summary linked to this article.

## Supplementary information

Supplementary Information Supplementary Figs. 1–10 and their legends.

Reporting Summary

Supplementary Tables 1–11

## Source data

Source Data Fig. 1 Statistical source data for Fig. 1.

Source Data Fig. 2 Statistical source data for Fig. 2.

Source Data Fig. 3 Statistical source data for Fig. 3.

Source Data Fig. 4 Statistical source data for Fig. 4.

Source Data Fig. 6 Statistical source data for Fig. 6.

Source Data Fig. 7 Statistical source data for Fig. 7.

Source Data Fig. 8 Statistical source data for Fig. 8.

Source Data Extended Data Fig. 1 Statistical source data for Extended Data Fig. 1.

Source Data Extended Data Fig. 2 Statistical source data for Extended Data Fig. 2.

Source Data Extended Data Fig. 3 Statistical source data for Extended Data Fig. 3.

Source Data Extended Data Fig. 4 Statistical source data for Extended Data Fig. 4.

Source Data Extended Data Fig. 5 Statistical source data for Extended Data Fig. 5.

Source Data Extended Data Fig. 6 Statistical source data for Extended Data Fig. 6.

Source Data Extended Data Fig. 8 Statistical source data for Extended Data Fig. 8.

Source Data Extended Data Fig. 9 Statistical source data for Extended Data Fig. 9.

Source Data Extended Data Fig. 10 Statistical source data for Extended Data Fig. 10.

Source Data Extended Data Fig. 2 Unprocessed blot for Extended Data Fig. 2.

## Data Availability

Bulk RNA-seq, snRNA-seq and spatial transcriptomic data that support the findings of this study have been deposited in the Gene Expression Omnibus (GEO) under accession codes GSE246251 and GSE246770. Human prostate cancer bulk RNA-seq data were derived from ref. ^48^ and downloaded from GitHub at https://github.com/cBioPortal/datahub/tree/master/public/prad_su2c_2019. Publicly available mouse and human single-cell RNA-seq datasets were used and can be found in refs. ^16,80^ and under GEO accession numbers GSE210358 and GSE264573, respectively. CRISPR-targeted locus sequencing datasets have been submitted to the Sequence Read Archive and are available under BioProject ID PRJNA1031236 at https://www.ncbi.nlm.nih.gov/bioproject/PRJNA1031236. Uncropped western blots have been provided as an Extended Source Data file. Source data for Figs. 1–4 and 6–8 and Extended Data Figs. 1–6 and 8–10 have been provided as Source Data files. Segmented (HALO) mIF (7-plex) tumor-cell-centric datasets from *Ascl1*^wt^ and *Ascl1*^KO^ RPM tumors have been uploaded to figshare at 10.6084/m9.figshare.c.7470099.v1 (ref. ^81^). TME segmentation data (COMET) in this study cannot be deposited in a public repository due to size constraints but are available from the corresponding author upon request. All other data supporting the findings of this study are available from the corresponding author upon request. Requests will be processed within 14 days. Source data are provided with this paper.

## Code availability

Bulk RNA-seq analysis, spatial transcriptomics and spatial immunofluorescence analyses were performed using open-source software as described in Methods. Available code from these analyses has been deposited at GitHub at https://github.com/igordot.sns, https://github.com/dpeerlab/PrismSpot and figshare at 10.6084/m9.figshare.c.7470099.v1 (ref. ^81^).
