# Supplementary material for: The neuroendocrine transition in prostate cancer is dynamic and dependent on ASCL1
Source: Nat Cancer. 2024 Oct 11;5(11):1641–59. doi: 10.1038/s43018-024-00838-6 (PMC11584404; doi:10.1038/s43018-024-00838-6)
Supplement: Supplementary file 1 — Supplementary Figs. 1–10 and their legends. [file 43018_2024_838_MOESM1_ESM.pdf]

nature cancer

Supplementary information

https://doi.org/10.1038/s43018-024-00838-6

The neuroendocrine transition in prostate cancer is dynamic and dependent on ASCL1

In the format provided by the authors and unedited

SUPPLEMENTARY FIGURE 1:

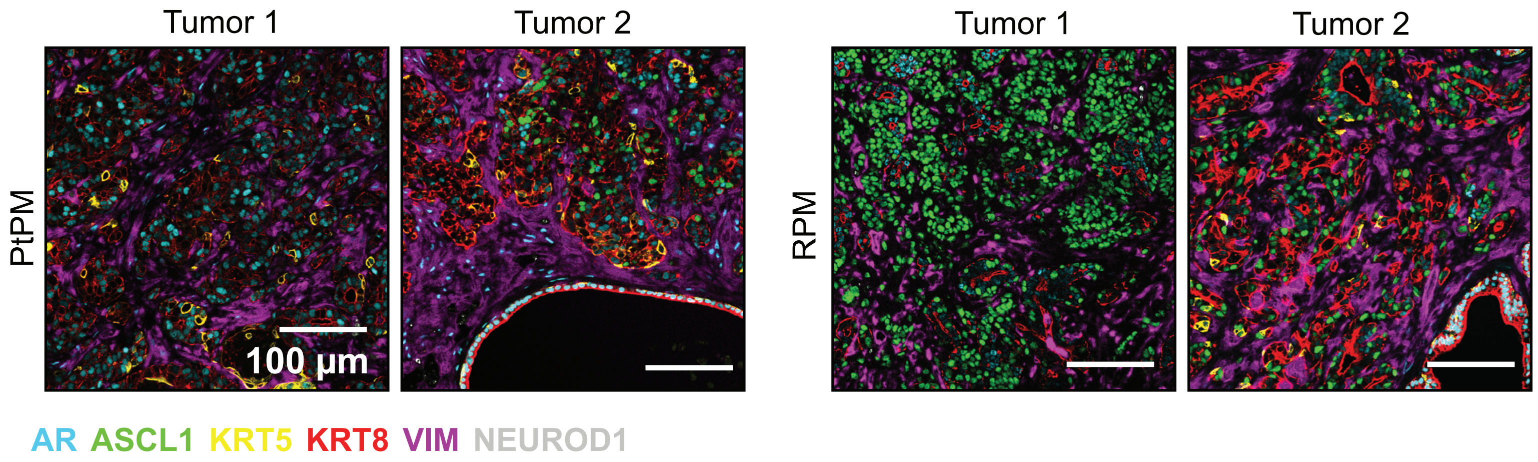

Supplementary Figure 1: *Rb1* loss sensitizes prostate tumors for the neuroendocrine transition

Representative multiplexed immunofluorescence images, n = 2 tumor samples (3-5 weeks post transplantation) for each genotype. Images related to Extended Data Fig. 2j-l. Data representative of n = 4 tumors (PtPM) and n = 5 tumors (RPM).

SUPPLEMENTARY FIGURE 2:

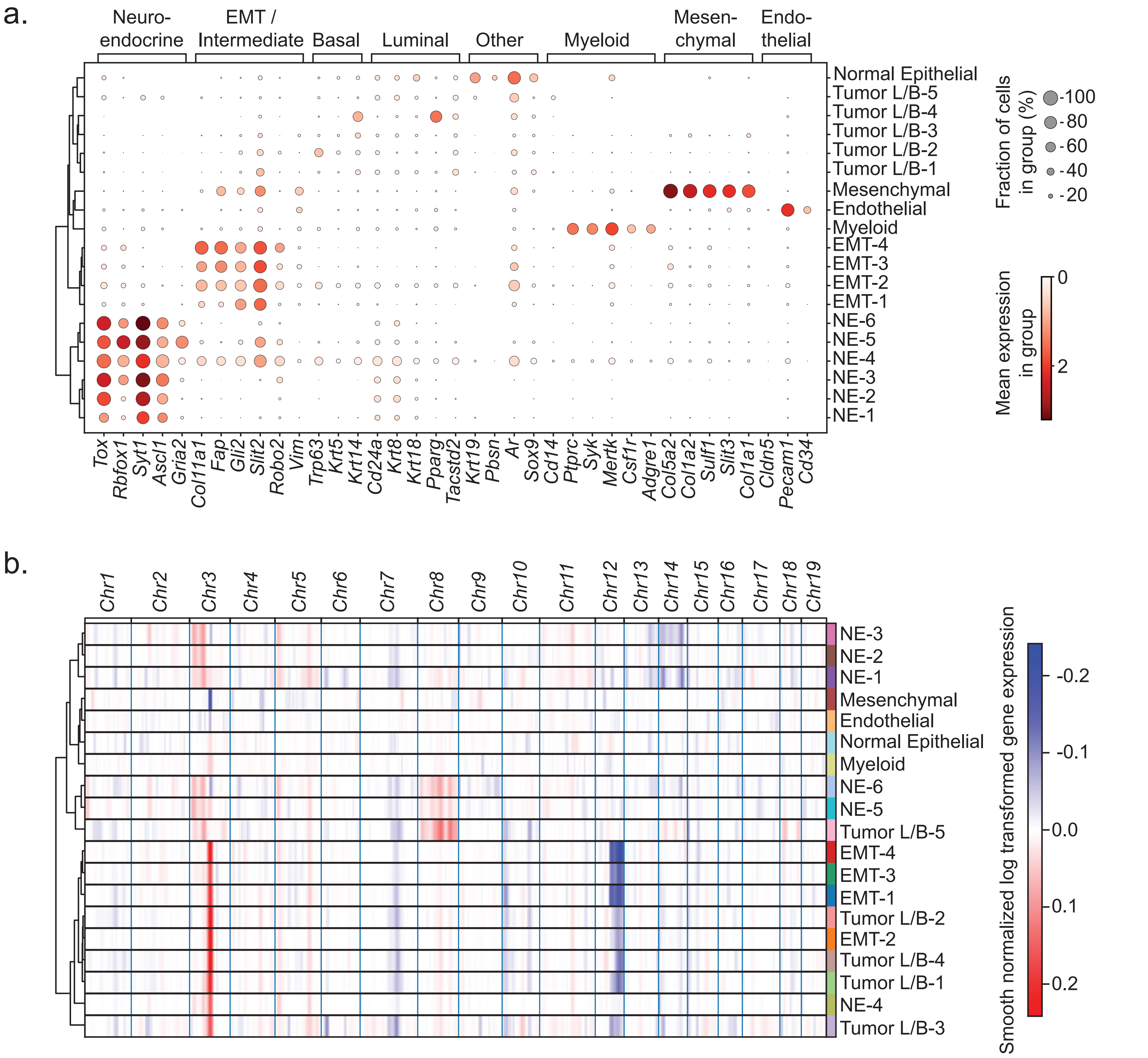

Supplementary Figure 2: Single nuclear RNAseq cell typing used as input for Visium analysis.

a. Dot plot of selected marker genes (DEGs) associated with each of the indicated cell types from snRNA-seq profiled from RPM tumors. Data represent the mean normalized $log_2(X+1)$ expression scaled from 0 to 1. Dot size represents percent of cells expressing a given gene.

b. Inferred copy number variation (CNV) applying inferCNV to snRNAseq profiled from RPM tumors. Data represent the smooth normalized and log-transformed gene expression values across each murine chromosome. Inferred CNV for each cell type identified in panel a listed on the right.

SUPPLEMENTARY FIGURE 3:

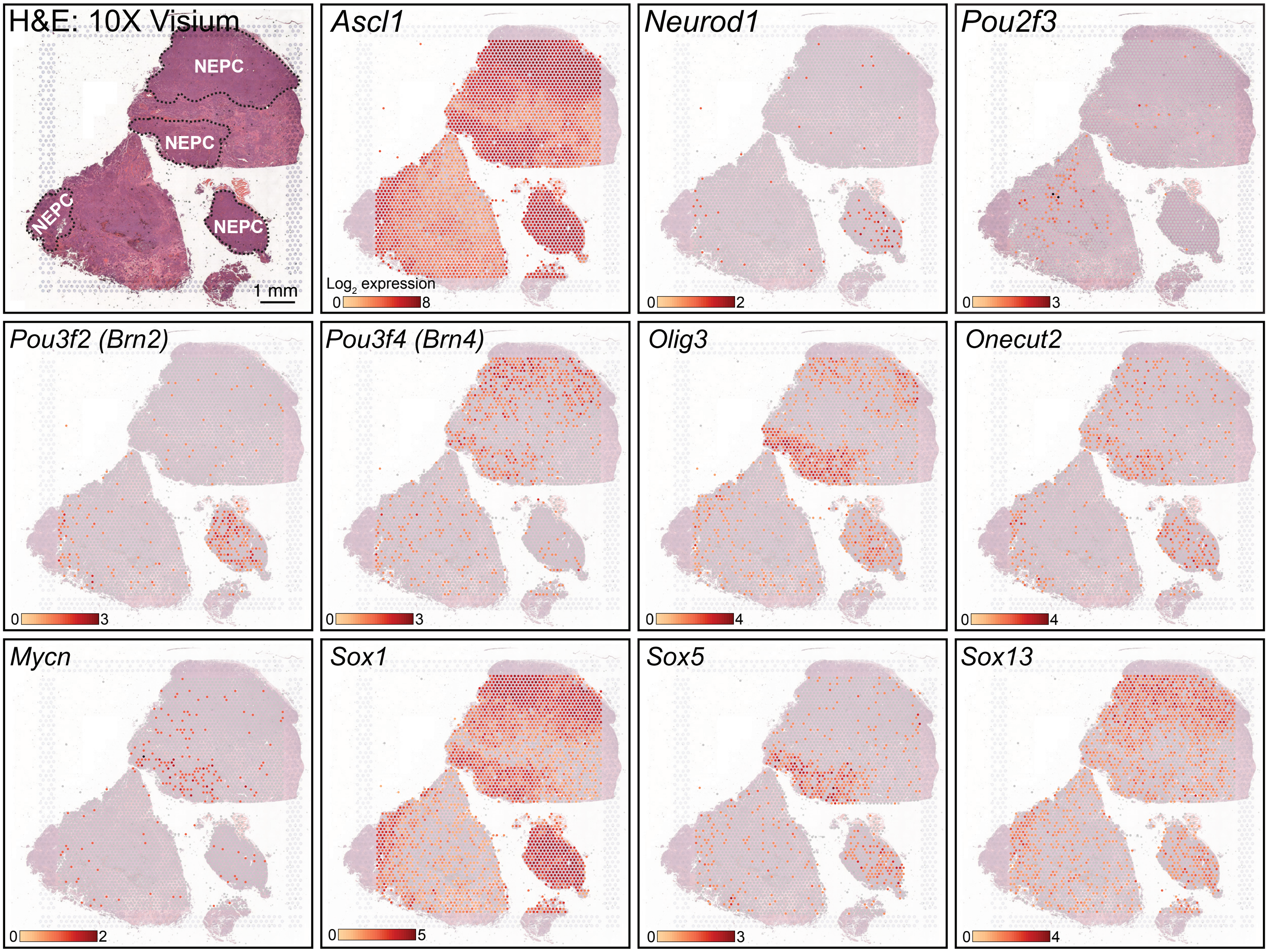

Supplementary Figure 3: Spatial hetereogeneity in TF expression

Log_2 spatial expression (Visium) across the indicated genes. Top Left: H&E depicting NEPC tumor regions within two independent 10-week RPM tumors (related to Fig. 5c).

SUPPLEMENTARY FIGURE 4:

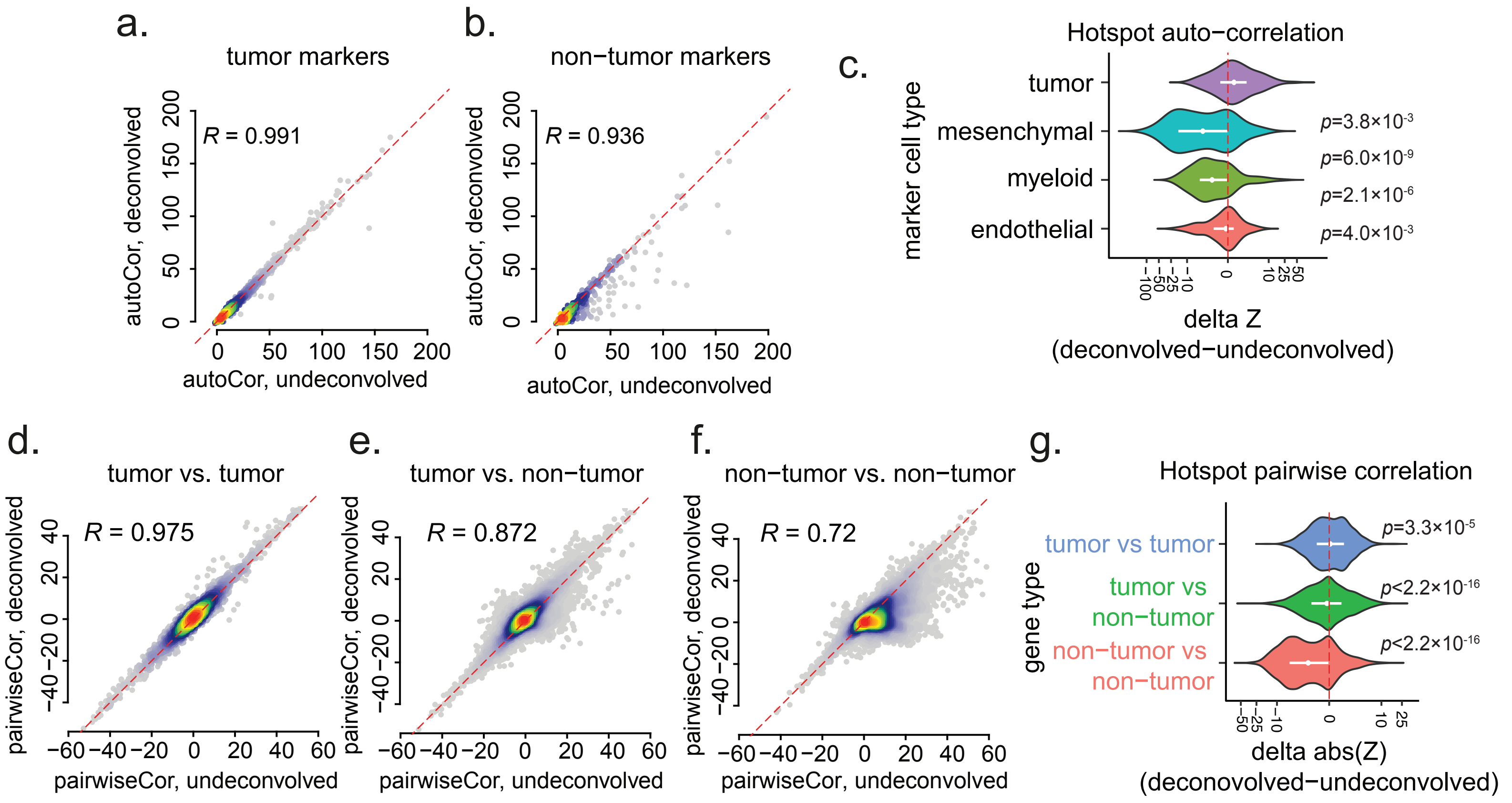

Supplementary Figure 4: PrismSpot demonstrates stronger signal to noise for tumor and non-tumor-specific gene modules

a. Density plot shows the Hotspot autocorrelation *Z*-scores for BayesPrism deconvolved (y-axis) vs undeconvolved expression (x-axis) across markers of tumor cells derived from GEMM scRNA-seq. Each dot in the density plot represents a tumor marker gene. Red dashed line marks y=x. **b.** Same as panel a. but across markers of non-tumor cell types derived from GEMM scRNA-seq, including endothelial, myeloid, and mesenchymal cells. Autocorrelation computed using the deconvolved expression shows smaller value than that from the undeconvolved expression. **c.** Violin plot shows the distribution of the difference between *Z*-scores of PrismSpot and un-deconvolved Hotspot for marker genes of each cell type. Red dashed line marks zero. **d.** Density plot shows the Hotspot pairwise local correlation *Z*-scores for BayesPrism deconvolved (y-axis) versus undeconvolved expression (x-axis) between pairs of tumor marker genes defined using GEMM scRNA-seq. Each dot in the density plot represents a pair of tumor marker genes. Red dashed line marks y=x. **e.** Same as panel d. but between a tumor marker gene and marker gene from any non-tumor cell types. Using deconvolved expressions shrinks local correlation *Z*-scores towards zero. **f.** Same as panel d. but between a pair of marker genes from any non-tumor cell types. Using deconvolved expressions shrinks local correlation *Z*-scores towards zero. **g.** Violin plot shows the distribution of the difference between the absolute value of *Z*-scores of PrismSpot and un-deconvolved Hotspot for genes of each category. Red dashed line marks zero.

SUPPLEMENTARY FIGURE 5:

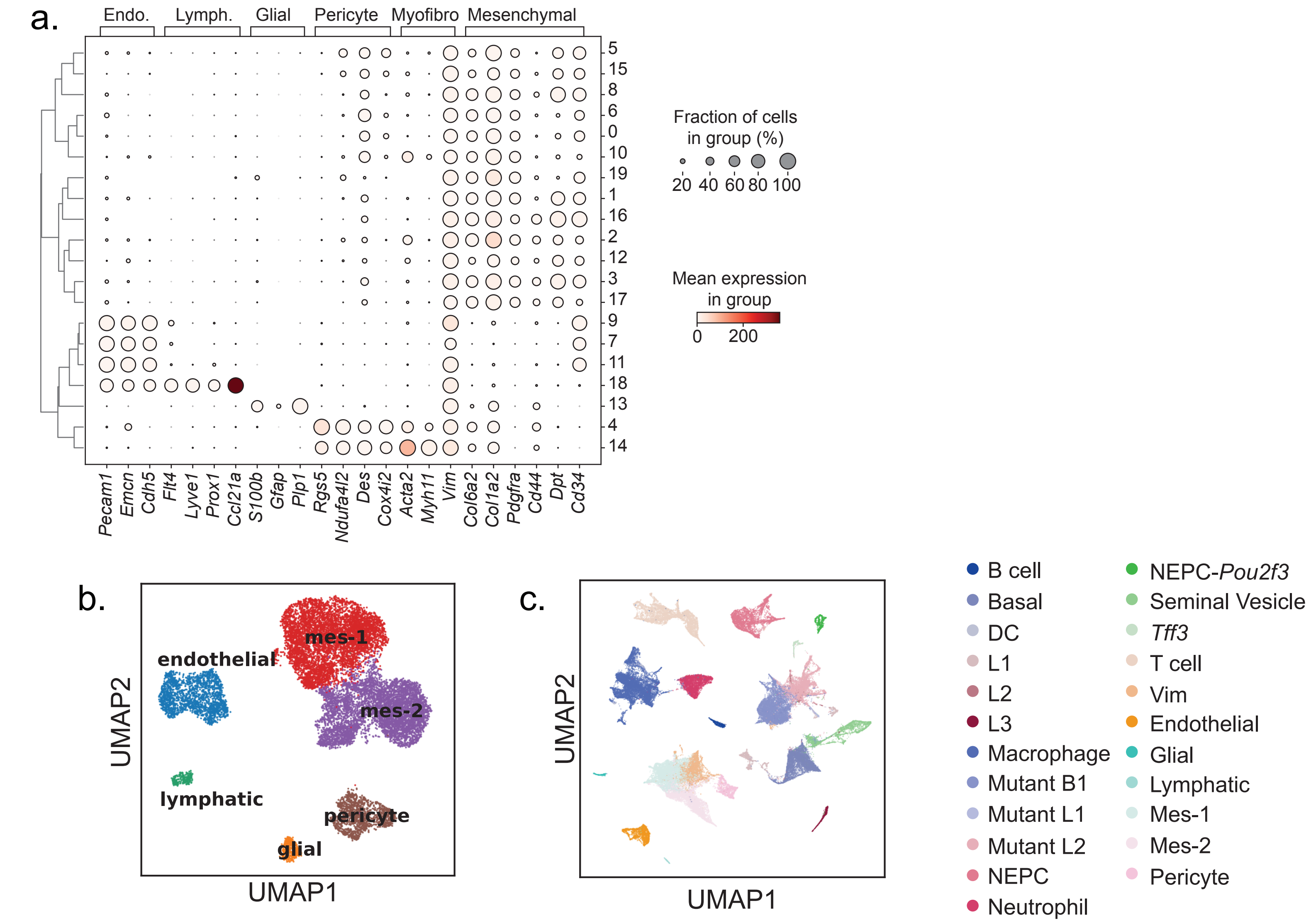

Supplementary Figure 5: Non-tumor cell typing from previously published datasets used as input for BayesPrism analysis of Visium dataset.

a. Dot plot of marker genes associated with each of the indicated cell types across phenograph clusters. Data represent the mean normalized $log_2(X+1)$ expression scaled from 0 to 1. Dot size represents percent of cells expressing a given gene.

b. UMAP of non-tumor cell populations in GEMM scRNA-seq dataset, colored by cell types. Mes = Mes-enchymal.

c. UMAP of the original GEMM scRNA-seq dataset, colored by cell types with the GFP-negative stromal populations replaced with finer-grained cell types. Data derived from *n* = 13 “PtRP” mice.

SUPPLEMENTARY FIGURE 6:

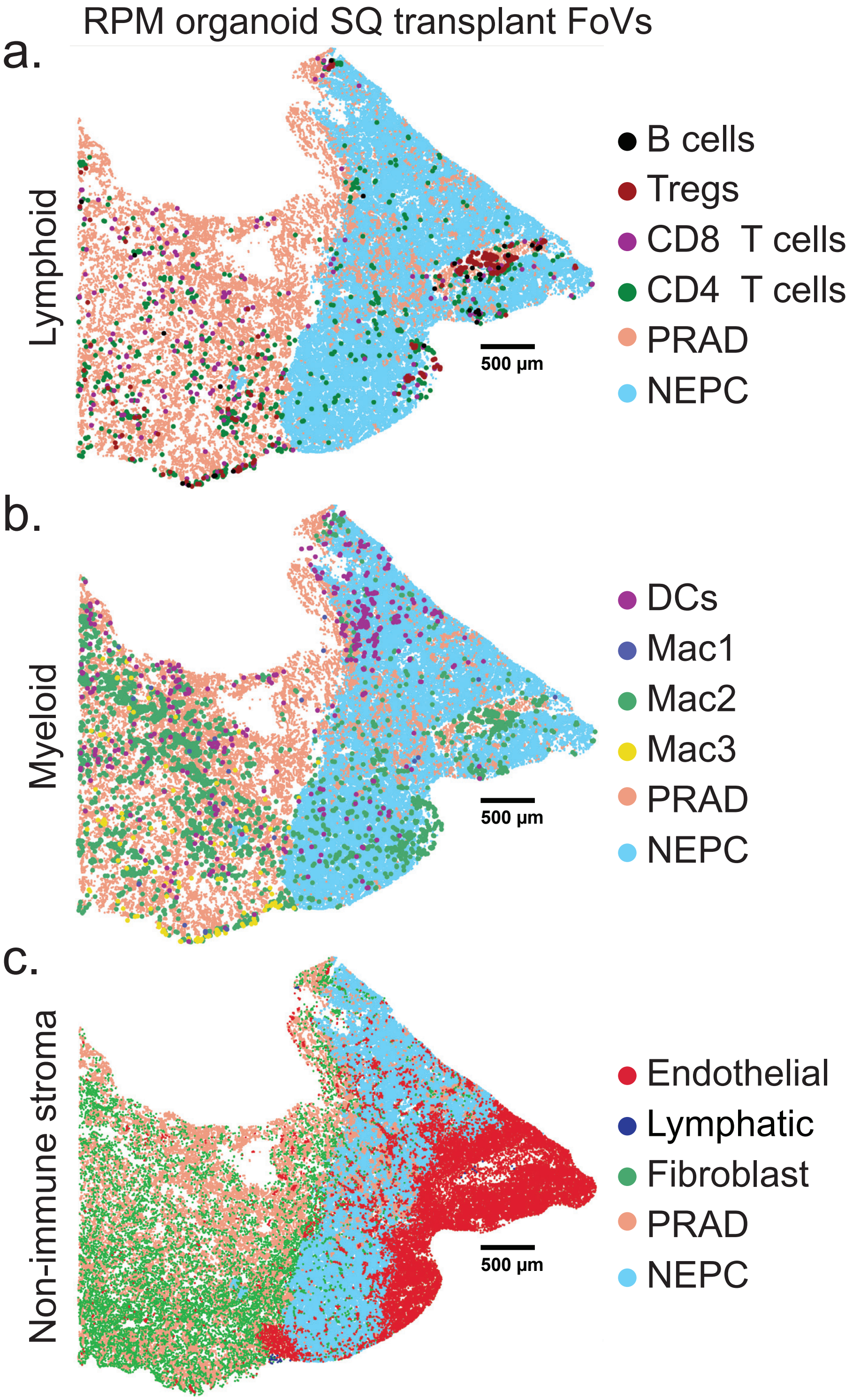

Supplementary Figure 6: TME in flank RPM tumors is similar to RPM orthotopic tumors. Representative segmented field of view (FoV) for the indicated general **a.** lymphoid cell, **b.** Myeloid, and **c.** non-immune stromal types in 8-week SQ RPM tumor. Data are representative of *n* = 5 tumors. SQ = subcutaneous tumor.

SUPPLEMENTARY FIGURE 7:

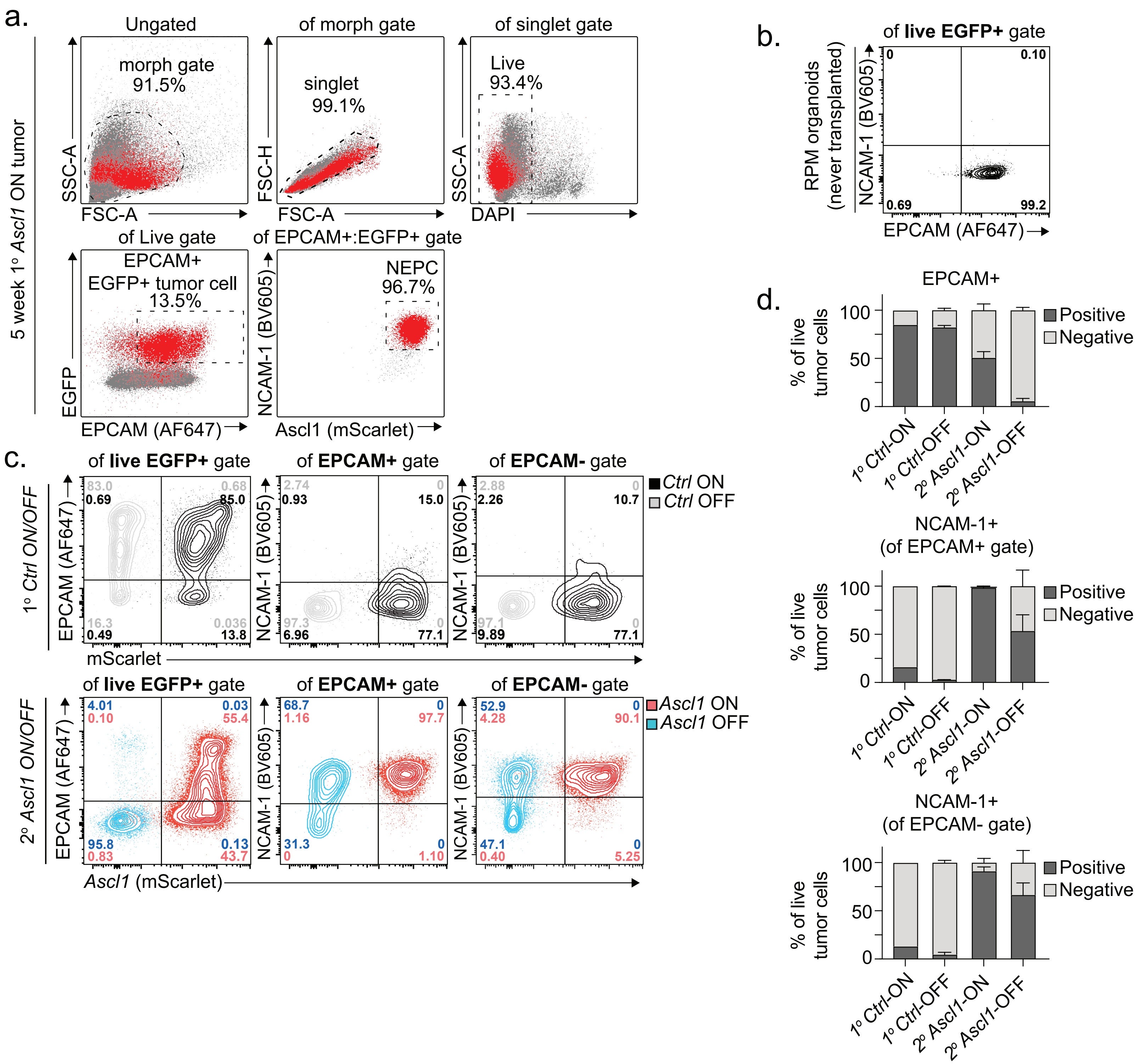

Supplementary Figure 7: *Ascl1* loss in estbalished NEPC results in loss of EPCAM.

a. Gating strategy used to purify RPM-NEPC tumor fraction from primary (1º) *Ascl1 ON* mice on dox for 5 weeks post transplantation. Data related to Fig. 8c. See methods for more information.

b. Representative flow plot of control (DAPI-negative) RPM organoids (never transplanted, EGFP+) stained with NCAM-1 or EPCAM and used for positive and negative gating in panels c-d.

c. (Top) Representative OT *Ctrl ON* or *Ctrl OFF* primary (1º) tumors (endogenous *Ascl1*^{KO}, can only initiate and progress as PRAD) stained for the depicted markers. Used as negative controls for NCAM-1 staining. (Bottom) Representative SQ *Ascl1 ON* or *Ascl1 OFF* secondary (2º) tumors stained for the depicted markers. Outliers marked as dots. Note 95.8% of *Ascl1 OFF* tumors (dox withdrawal for 2 weeks) have lost EPCAM expression.

d. Stacked bar charts for the indicated tumors staining positively or negatively for (top) EPCAM, (middle) NCAM-1 of EPCAM+ gate, or (bottom) NCAM-1 of EPCAM- gate. Related to panel c. Error bars represents mean and standard deviation and data from panels c-d are derived from $n = 5$ tumors per group.
