# Supplementary material for: The neuroendocrine transition in prostate cancer is dynamic and dependent on ASCL1
Source: Nat Cancer. 2024 Oct 11;5(11):1641–59. doi: 10.1038/s43018-024-00838-6 (PMC11584404; doi:10.1038/s43018-024-00838-6)
Supplement: Supplementary file 2 — Reporting Summary [file 43018_2024_838_MOESM2_ESM.pdf]

nature portfolio

Corresponding author(s): Charles L. Sawyers

Last updated by author(s): Aug 31, 2024

Reporting Summary

Nature Portfolio wishes to improve the reproducibility of the work that we publish. This form provides structure for consistency and transparency in reporting. For further information on Nature Portfolio policies, see our Editorial Policies and the Editorial Policy Checklist.

Statistics

For all statistical analyses, confirm that the following items are present in the figure legend, table legend, main text, or Methods section.

n/a	Confirmed	
☐	☒	The exact sample size (*n*) for each experimental group/condition, given as a discrete number and unit of measurement
☐	☒	A statement on whether measurements were taken from distinct samples or whether the same sample was measured repeatedly
☐	☒	The statistical test(s) used AND whether they are one- or two-sided<br>*Only common tests should be described solely by name; describe more complex techniques in the Methods section.*
☐	☒	A description of all covariates tested
☐	☒	A description of any assumptions or corrections, such as tests of normality and adjustment for multiple comparisons
☐	☒	A full description of the statistical parameters including central tendency (e.g. means) or other basic estimates (e.g. regression coefficient) AND variation (e.g. standard deviation) or associated estimates of uncertainty (e.g. confidence intervals)
☐	☒	For null hypothesis testing, the test statistic (e.g. *F*, *t*, *r*) with confidence intervals, effect sizes, degrees of freedom and *P* value noted<br>*Give P values as exact values whenever suitable.*
☐	☒	For Bayesian analysis, information on the choice of priors and Markov chain Monte Carlo settings
☒	☐	For hierarchical and complex designs, identification of the appropriate level for tests and full reporting of outcomes
☐	☒	Estimates of effect sizes (e.g. Cohen's *d*, Pearson's *r*), indicating how they were calculated

Our web collection on statistics for biologists contains articles on many of the points above.

Software and code

Policy information about availability of computer code

Data collection

Flow cytometry data were acquired by using the Sony Cell Sorter Software (v3.2). Applied Biosystems QuantStudio 6 (v1.3) was used for real-time PCR. Western blot imaging acquisition was performed by using ImageQuant 800 Control Software (v1.2.0) GE Health Care Life Science Technologies. Mulitplexed immunofluorescence stains were obtained with the Lunaphore COMET software (v1.1.0.0). Tumor volume by ultrasound imaging was collected by using Vevo2100 (Vevo LAB 5.7.1) from VisualSonics, Inc. Microscopy images were collected on a Pannoramic Scanner (3DHistotech). Live organoid fluorescence images were collected with NIS-Elements AR (v5.21.03). Stereoscopic images collected with NIS-Elements BR (v.5.31.01). Confocal images collected with LAS (v4.5.0.25531).

Data analysis

We used GraphPad Prism software v.9.5.1 for statistical analyses or in-house scripts in R v.4.3.1 which are available from the corresponding author upon reasonable request. All other relevant code is found deposited on github (see links below).

Vevo Lab Software (v5.9.0)
FlowJo (build 10.4.2)
ImageJ2 (v2.9.0/1.53t, build a33148d777)
QuPath (v0.4.2)
Prism (v.10.0.3)
CRISPresso2 (http://crispresso.pinellolab.org/)
MultiQC (python/cpu/v2.7.15)
Trimmomatic (v0.36)
splice-aware (STAR v2.7.3a)
featureCounts (subread/v1.6.3)
DESeq2 (r/v4.1.2)

pheatmap (v1.0.12)
ComplexHeatmap (v2.16.0)
EnhancedVolcano (v.1.18.0)
fgsea (v1.26.0)
msigdbr (v.7.5.1)
GSEA desktop application (v4.3.2)
10X Cell Ranger (v6.1.2 and v7.0.1)
10X spaceranger (v2.0.0)
HALO (Indica Labs, v3.6.4124)
Cell Bender (https://github.com/broadinstitute/CellBender)
Scanpy (https://github.com/scverse/scanpy)
Scrublet (https://github.com/swolock/scrublet)
Phenograph (https://github.com/jacoblevine/PhenoGraph)
InferCNV (https://github.com/broadinstitute/inferCNV)
BayesPrism (https://github.com/Danko-Lab/BayesPrism)
Hotspot (https://github.com/YosefLab/Hotspot)
Mesmer (https://github.com/vanvalenlab/deepcell-tf)

Code for bulk RNA-sequencing available on Github: https://github.com/igordot/sns
Code for PrismSpot analysis available on Github: https://github.com/dpeerlab/PrismSpot
Code for Mesmer analysis available on Figshare: https://figshare.com/articles/journal_contribution/Source_code_used_for_Fig_4_Mesmer_segmentation_/26400259?file=48003763

For manuscripts utilizing custom algorithms or software that are central to the research but not yet described in published literature, software must be made available to editors and reviewers. We strongly encourage code deposition in a community repository (e.g. GitHub). See the Nature Portfolio guidelines for submitting code & software for further information.

Data

Policy information about availability of data

All manuscripts must include a data availability statement. This statement should provide the following information, where applicable:

- Accession codes, unique identifiers, or web links for publicly available datasets
- A description of any restrictions on data availability
- For clinical datasets or third party data, please ensure that the statement adheres to our policy

In-house mouse bulk RNAseq, snRNAseq and spatial transcriptomic data that support the findings of this study have been deposited in the Gene Expression Omnibus under accession codes GSE246251 and GSE246770. Mouse reference genome assembly GRCm30/mm10 was accessed from NCBI (refseq assembly GCF_000001635.27). Human prostate cancer bulk RNA-seq dataset were derived from ref.49 and was downloaded from github via the hyperlink: https://github.com/cBioPortal/datahub/tree/master/public/prad_su2c_2019. Publicly available mouse and human single cell RNA-seq datasets were used and can be found in refs.16,79, under accession numbers GSE210358 and GSE264573, respectively. CRISPR targeted locus sequencing datasets have been submitted to the Sequence Read Archive and are available under BioProject ID PRJNA1031236. Uncropped western blots have been provided as Source Extended Data Fig 2. Source data for Main and Extended Data Figures are provided as Source Data files. All other data supporting the findings of this study are available from the corresponding author on reasonable request. Requests will be processed within 14 days.

Research involving human participants, their data, or biological material

Policy information about studies with human participants or human data. See also policy information about sex, gender (identity/presentation), and sexual orientation and race, ethnicity and racism.

Reporting on sex and gender	As outlined in the methods, tumor samples were collected at MSKCC from a 62-year-old male with localized PRAD undergoing XRT followed by salvage prostatectomy post ADT and docetaxel. Tumor in the bladder arose by extension of a prostate tumor recurrence in the surgical bed. No gender related issues are applied to this analysis.<br><br>Since the study involved only a single male patient, gender-related factors were not considered in the analysis. The sex of the participant was self-reported, and no transgender individuals were included in this study.
Reporting on race, ethnicity, or other socially relevant groupings	Information regarding the race, ethnicity, or socially relevant grouping was not collected for the above listed patient.
Population characteristics	Tumor sample was obtained as described above.
Recruitment	Tumor material was collected from tumor that arose in the bladder by extension of a prostate tumor recurrence in the surgical bed.
Ethics oversight	This study was approved by MSKCC's (New York, NY, USA) Institutional Review Board (IRB) #21-005 (NCT: 01775072). Patient gave informed consent.

Note that full information on the approval of the study protocol must also be provided in the manuscript.

Field-specific reporting

Please select the one below that is the best fit for your research. If you are not sure, read the appropriate sections before making your selection.

☒ Life sciences ☐ Behavioural & social sciences ☐ Ecological, evolutionary & environmental sciences

For a reference copy of the document with all sections, see nature.com/documents/nr-reporting-summary-flat.pdf

Life sciences study design

All studies must disclose on these points even when the disclosure is negative.

Sample size

No formal sample size calculation was performed. However, the sample sizes used are consistent with those reported in similar experiments and publications (Chan et al., Cancer Cell, 2021; Chan et al., Science, 2022; Niec et al., Cell Stem Cell, 2022). Exact sample sizes are provided in the figure legends. For all experiments, at least three replicates or animals per group were used to determine statistical significance. All cell-based assays were conducted in independent experiments, with cells infected and seeded into technical replicates. Any data from a single experiment are noted in the figure legends, along with a statement on reproducibility.

For animal studies, more than three mice were used per experimental cohort for each genotype. Tumor mass, volume, and histopathological analyses were conducted on more than three mice per genotype. Sample sizes for mice were chosen based on the type of experiment. For subcutaneous allografts, more than three mice with one to two tumors per mousse were used. For orthotopic transplantation of organoids, two separate cohorts of more than five mice per group were used, and results from both experiments were combined for analysis.

For all cell line or organoid experiments, as previously stated, data were derived from more than three independent experiments unless otherwise noted. Sample size was selected due to the low inherent variability and high reproducibility between experiments.

Data exclusions

Animals lacking detectable tumors by histopathology or animals with severely ulcerated tumors prior to experimental end-point were excluded from the analysis to ensure all animals were successfully transplanted with tumor cells. No other data were excluded from any analysis.

Replication

All experiments were replicated two or more times using the same experimental approach. For mouse and cell experiments, two or more sgRNAs targeting distinct sequences of the gene of interest were used.

For mouse tumor analysis, the total number of mice shown in the representative figures was derived from two separate cohorts transplanted at different times but sacrificed at equivalent time points post-infection and tumor induction.

For RT-qPCR analysis of Ascl1 expression across organoids and isolated RPM tumors, four technical replicates were used, and the experiment was successfully repeated. Primer sequences used for qPCR are provided in Supplementary Table 11.

For multiplexed immunofluorescence (IF), three or more tumors (from independent mice) were analyzed. For metastasis studies, all tumor nodules within a single tissue section were analyzed to obtain an average infiltration score per cell type for each metastatic sample.

Randomization

Only male mice aged 8-12 weeks with the correct genotypes were randomly selected for tumor transplantation studies. Male mice were used to maintain the testosterone environment necessary for appropriate tumor formation in this sex-specific context. Males were used in both subcutaneous and orthotopic transplantation assays. In vivo, animals were randomized into experimental groups based on similar tumor volumes measured by ultrasound or caliper. For in vitro studies, randomization was not possible; however, all cell lines/organoids were treated identically without prior designation.

Blinding

Investigators were blinded to group allocation during data collection and analysis. Tumor measurements, treatment administration, surgeries, and analyses were performed by different researchers to ensure blinding. Post-mortem tumor histopathological analysis was conducted in consultation with Dr. Anuradha Gopalan, a genitourinary pathologist, who was blinded to the genotype of the samples.

Ultrasound and caliper measurements were performed under blinded conditions using mouse ID. All burden analysis and immunohistochemistry (IHC) were also conducted in a blinded manner. More information can be found in the Methods section.

Reporting for specific materials, systems and methods

We require information from authors about some types of materials, experimental systems and methods used in many studies. Here, indicate whether each material, system or method listed is relevant to your study. If you are not sure if a list item applies to your research, read the appropriate section before selecting a response.

Materials & experimental systems

n/a	Involved in the study	
☐	☒	Antibodies
☐	☒	Eukaryotic cell lines
☒	☐	Palaeontology and archaeology
☐	☒	Animals and other organisms
☒	☐	Clinical data
☒	☐	Dual use research of concern
☒	☐	Plants

Methods

n/a	Involved in the study	
☒	☐	ChIP-seq
☐	☒	Flow cytometry
☒	☐	MRI-based neuroimaging

Antibodies

Antibodies used	Antibody information and dilutions used in this manuscript for IHC, IF, western blotting, and flow cytometry are listed in Supplementary Table 10.<br><br>Antibodies and dilutions used in the Lunaphore COMET assays are found in Supplementary Table 5.
Validation	All antibodies used in this study had been previously published or validated using engineered knock-out or shRNA-mediated knock-down cell lines or cross validated against tissues harboring cells with known marker expression. The antibodies used are established in the field and have been used by a number of other groups. Some anitbodies have been additionally validated for use in their respective application. For Western blot, KO cells for Ascl1 were used as negative controls. Overexpressing cells for Ascl1 was used as a positive control. For histology and immunofluorescence, as a negative control one section per slide was stained following the same protocol and omitting the primary antibody or negative and positive control tissues were used. For flow cytometric analysis and FACS, FMO and isotype controls were evaluated for each antibody ensure specificity across all staining parameters.

Eukaryotic cell lines

Policy information about cell lines and Sex and Gender in Research

Cell line source(s)	293T was obtained from Takara Bio (#632180). All other cell lines used in this study were established as part of the study. All organoid lines were created and established from one or more C57BL/6J male hosts as indicated in the methods.
Authentication	No authentication was used.
Mycoplasma contamination	All cell lines used in this study were regularly tested for Mycoplasma contamination every 6 months. All cells used in this study tested negative for Mycoplasma.
Commonly misidentified lines<br>(See ICLAC register)	No commonly misidentified cell lines were used in the study. Engineered prostate organoids generated in this study are not registered on ICLAC.

Animals and other research organisms

Policy information about studies involving animals; ARRIVE guidelines recommended for reporting animal research, and Sex and Gender in Research

Laboratory animals	Male mice were maintained under pathogen-free, temperature controlled (20-25 C), and a 12-hour light-dark cycle with 30-70% relative humidity. Food and water were provided ad libitum. Male mice, 8-12 weeks old, used for transplantation were maintained on a C57BL/6J background. Transplantation into immunocompetent hosts were performed on mice harboring conditional EGFP alleles to tolerize against EGFP-derived antigens expressed within organoids (Jax, #026179). Transplantation of organoids harboring dox-inducible constructs into immunodeficient hosts were performed in PrkcdKO mice (Jax, #001913) to avoid rtTA-mediated rejection. All mice received pre- and post-operative analgesia with meloxicam and buprenorphine and were monitored for any signs of discomfort or distress. At established experimental end-point, mice were euthanized by CO2 asphyxiation followed by cervical dislocation.
Wild animals	No wild animals were used in this study.
Reporting on sex	Findings within this manuscript are applicable only to the male sex. All experimental mouse models or recipients used in this study were male.
Field-collected samples	No samples were collected in the field.
Ethics oversight	All animal studies and procedures were approved by the MSKCC Institutional Animal Care and Use Committee (protocol # 06-07-012). MSKCC guidelines for the proper and human use of animals in biomedical research were followed. Maximal tumor burden permitted by MSKCC IACUC of 2cm^3 was not exceeded in this study.

Note that full information on the approval of the study protocol must also be provided in the manuscript.

Plants

Seed stocks	Seeds were not used in this study.
Novel plant genotypes	Plants were not used in this study.
Authentication	N/A

Flow Cytometry

Plots

Confirm that:

☒ The axis labels state the marker and fluorochrome used (e.g. CD4-FITC).

☒ The axis scales are clearly visible. Include numbers along axes only for bottom left plot of group (a 'group' is an analysis of identical markers).

☒ All plots are contour plots with outliers or pseudocolor plots.

☒ A numerical value for number of cells or percentage (with statistics) is provided.

Methodology

Sample preparation	Tumor-bearing mice were sacrificed by CO2 asphyxiation followed by cervical dislocation. Tumors from either primary or secondary RPM transplanted hosts were minced and dissociated in 2 mg/mL of Collagenase II (Life Technologies, 17101015) in ADMEM (Thermo, #12634028) supplemented with 1X Glutamax (Thermo, #35050079), 10 mM HEPES, 0.5X PenStrep (Thermo, #15140163), and 100 µg/mL Primocin (InvivoGen, ant-pm-1) for 20 minutes in a 37°C shaker. Cell suspensions were then passed through a 100 micron strainer and washed in 0.5% BSA in PBS twice and centrifuged for 5 min at 400g. For secondary transplanted tumors, 5-week Ascl1ON tumors were harvested and stained with NCAM-1 (BV605, BD, 748097, 1:250), EPCAM (AF647, Biolegend, 118212, 1:500) and DAPI, and sorted for DAPI-, EGFP+, mScarlet+, EPCAM+, NCAM-1+ cells (see Supplementary Table 10) in FACS buffer (0.5% BSA, 1 mM EDTA, 1X PBS) for 1 hour on ice. Stained single cell suspensions were subsequently washed 3X (5 min 400g) in FACS buffer, strained, and processed for flow cytometry. Control tissues (primary control tumors lacking Ascl1 expression but positive for mScarlet) or never transplanted RPM organoids were used to confirm gating parameters for EGFP, mScarlet, EPCAM, and NCAM-1 including FMO controls.
Instrument	Sony MA900 (Sony Biotechnology) with a 130-µm sorting chip (Sony Biotechnology, #LE-C3213).
Software	Flow cytometry data collection was done with Sony Cell Sorter Software (v3.2)<br>Flow cytometry analysis was done with the FlowJo software v10.4.2)
Cell population abundance	Relative abundance of each gated population can be found in gating strategy summary shown in Supplementary Figure 7a. Sample purity was not evaluated prior to transplantation into secondary hosts.
Gating strategy	Cell subtypes were gated as follows: Live NEPC tumor cells were gated for FSC/SSC, EGFP+, EPCAM+, NCAM-1+, and mScarlet +. All gatings were performed on the viable cell fraction, excluding cells positive for DAPI. Gating strategy information can be found in Supplementary Fig. 7a.

☒ Tick this box to confirm that a figure exemplifying the gating strategy is provided in the Supplementary Information.
